# T cell and monocyte activation in concert with hematopoietic stem cell interactions shapes the post-allogeneic transplant immune landscape in poor graft function

**DOI:** 10.3389/fimmu.2026.1750093

**Published:** 2026-02-04

**Authors:** Ashvind Prabahran, Zhijie Wu, Shouguo Gao, Huw Morgan, Nicholas Holzwart, Mandy Ludford-Menting, Mayani Rawicki, Jessica Klass, Ray-Mun Koo, Clarissa Wilson, Piers Blombery, Chin Wee Tan, Saanvi Indukuri, Lynette Chee, David Ritchie, Neal S. Young, Xingmin Feng, Rachel Koldej

**Affiliations:** 1 Department of Clinical Haematology and Bone Marrow Transplantation, Peter MacCallum Cancer Centre and The Royal Melbourne Hospital, Melbourne, VIC, Australia; 2 Australian Cancer Research Foundation Laboratory, The Royal Melbourne Hospital, Melbourne, VIC, Australia; 3 Department of Medicine, The University of Melbourne, Melbourne, VIC, Australia; 4 Hematology Branch, National Heart, Lung, and Blood Institute, National Institutes of Health, Bethesda, MD, United States; 5 Department of Molecular Pathology, Peter MacCallum Cancer Center, Melbourne, VIC, Australia; 6 Walter and Eliza Hall Institute of Medical Research, Parkville, VIC, Australia

**Keywords:** hematopoietic cell transplantation, immune cell interactions, immune reconstitution, single-cell RNA sequence, T cell receptor

## Abstract

**Introduction:**

Post-allogeneic stem cell transplantation (alloSCT) can be complicated by poor graft function (PGF), a life-threatening condition characterized by complete donor chimerism alongside persistent multilineage cytopenias. PGF significantly increases the risk of bleeding, infection, and transfusion dependence. The cellular changes during hematopoiesis post-alloSCT, particularly in PGF, remain poorly defined.

**Methods:**

To evaluate the immune and hematopoietic reconstitution and dysfunction post-alloSCT, with a focus on PGF, we applied a comprehensive suite of histological, immunological, and molecular biological techniques to bone marrow (BM) and peripheral blood samples from patients with PGF, good graft function (GGF), and healthy donors (HDs).

**Results:**

By approximately 100 days post-alloSCT, patients demonstrated T cell oligoclonality, activation, and exhaustion compared to HDs. BM nucleated cells, particularly monocytes, exhibited increased activation and IFN-g response post-alloSCT compared to those of HDs. Moreover, cell-cell interactions between immune cells and hematopoietic stem and progenitor cells were notably enhanced post-alloSCT. While most inflammatory changes were present in both PGF and GGF, they were more pronounced in PGF.

**Discussion:**

Our results demonstrate a hyper-inflamed post-alloSCT environment involving both innate (monocytes) and adaptive (T cells) immune responses and their active interactions, more in PGF, highlighting that immune modulation may serve as an alternative or adjunctive therapeutic approach for PGF.

## Highlights

- Immune reconstitution is prolonged following hematopoietic cell transplantation, with hematopoietic stem and progenitor cell repopulation showing a slight myeloid bias.
- T cells remain highly clonally expanded approximately 3 months post-transplantation, exhibiting activated phenotypes, particularly in patients with poor graft function.
- Interactions among multiple immune cell types likely contribute to a hyperinflammatory bone marrow microenvironment following transplantation, a phenomenon that is exacerbated in poor graft function.

## Introduction

Allogenic stem cell transplantation (alloSCT), the transfer of hematopoietic stem cells (HSCs) from a donor into a compatible recipient, is a potentially curative therapy for a variety of hematologic disorders. A key indicator of transplantation success is the establishment of donor-derived hematopoiesis, assessed by normalization of peripheral blood (PB) counts and confirmation of complete donor chimerism through short tandem repeat analysis. Poor graft function (PGF) is a significant and potentially life-threatening complication of alloSCT, characterized by persistent multilineage cytopenias despite full donor chimerism (1). Patients with PGF are at increased risk for the consequences of bone marrow (BM) failure, including bleeding, infection, and dependence on frequent transfusions.

Our current understanding of hematopoiesis following alloSCT is primarily derived from experimental models and limited clinical data. Compared to polyclonal steady state hematopoiesis, post-alloSCT hematopoiesis is typically oligoclonal, driven by a few dominant HSC clones, which likely reflects both the selective mobilization of HSCs and the selective pressure imposed by inflammatory complications such as infection, and graft versus host disease (GVHD) (2, 3). BM functions post-alloSCT are characterized by a state of emergency hematopoiesis, with increased erythroid and myeloid differentiation (4), as well as elevated productions of inflammatory cytokines such as IFN- γ (5).

Related to hematopoiesis, immune reconstitution is an important process post-alloSCT and typically takes several months to fully establish. Successful immune reconstitution depends on various transplantation-related factors, including donor and recipient ages, graft sources, conditioning chemotherapy, and GVHD prophylaxis (6). The development of GVHD and post-transplantation complications, such as viral infections, can further impact immune reconstitution (7). At the cellular levels, different components of the immune system recover at distinct timepoints. Innate immune cells reconstitute rapidly, often within the first few weeks post-transplantation (8). In contrast, lymphopoiesis is a slower process, taking months to years. T-cell reconstitution, for example, initially relies on the peripheral expansion of donor-derived T cells, followed by *de novo* T-cell generation through BM lymphopoiesis (9). This gradual recovery is reflected in the T-cell receptor (TCR) repertoire, which is initially limited in diversity but expands over time.

The combination of the stressed hematopoietic system and limited immune repertoires post-alloSCT likely contributes to the development of PGF. At the cellular levels, several changes are associated with PGF, including alteration in the proportions of T-helper (Th)1 and Th17 cells, as well as the polarization of macrophages toward the M1 phenotype (10–12). Our group has utilized spatial proteomics to further investigate the BM microenvironment in patients with PGF, revealing upregulation of proteins such as CD44 and CD163. These proteins play roles in T-cell homing and macrophage activation, thus supporting an immunologic basis for the development of PGF (13). However, the exact mechanisms linking immune dysfunctions to stem cell suppression and BM failure in PGF remain under active investigation. Aplastic anemia (AA), a rare form of BM failure, is characterized by oligoclonal expansion of cytotoxic T cells and the apoptosis of HSCs. Immune mediated destruction in AA may share similarities with PGF. Indeed, previous studies have identified the emergence of PNH-type clones in PGF patients, akin to those observed in AA patients, suggesting potential shared underlying pathophysiology (14).

While previous studies have largely focused on individual cell populations and their dysfunctions in PGF, a more comprehensive investigation of hematopoietic and immune cell populations, as well as their specific interactions, particularly between immune cells and HSCs, is critical to uncover the mechanisms underlying BM failure. To better understand these critical cellular interactions and further characterize the immune dysregulation in PGF, we employed a comprehensive multiomic approach to evaluate both PB and BM samples from patients with PGF and those with good graft function (GGF) following alloSCT.

## Methods

### Patient samples

PB and BM samples were obtained from patients after written informed consent. PB samples from 14 healthy donors (HDs) were used as controls for flow cytometry analysis and were obtained from buffy-coat samples from the Australian Red Cross Blood Service. Bone marrow samples from four HDs (female/27, female/28, female/62, and male/23 years old) were used as controls for scRNA-seq analysis. Research was approved by the Institutional Review Board of the Royal Melbourne Hospital and NHLBI, in accordance with the Declaration of Helsinki. HDs for bone marrow samples were enrolled as controls under protocol NCT00001620 in NHLBI.

### Definitions of PGF and GGF

PGF patients were defined as follows 1) Complete myeloid donor chimerism, and 2) at least 2 lineage cytopenias defined as Hb<80g/L, Neutrophils <1.0x10^9^/L and Platelets <60x10^9^/L. GGF patients were defined as having complete donor chimerism and normal peripheral blood counts. with no evidence of relapse of their primary malignancy. PGF and GGF patients were matched for disease risk index, conditioning intensity, donor relation, graft type and cytomegalovirus (CMV) serostatus.

### Retrospective cohort

Recipients of alloSCT between 2017–2020 at Royal Melbourne Hospital Bone Marrow Transplant unit and alive to D60 were included in this analysis. Patients who died prior to D60 were excluded as chimerism data, which was required for our definition of PGF, was not often available prior to this date. The study was approved by the Melbourne Health Human Research Ethics committee and conducted in accordance with the Declaration of Helsinki. Survival analysis was performed in GraphPad Prism utilizing a Kaplan Meier Analysis.

### Bone marrow and peripheral blood samples processing

PB and BM specimens were obtained from patients and HDs. PB mononuclear cells (PBMCs) and BMMNCs were isolated using Ficoll-Paque Plus (GE Healthcare, Chicago, IL) and cryopreserved until used for flow cytometric and/or scRNA-seq.

### Flow cytometry of PBMCs and BMMNCs

Methods describing cell preparation, staining, acquisition, gating strategy, and analysis of PBMCs and BMMCs are detailed in supplementary information.

### Digital spatial profiling

DSP was performed as previously described (13). Briefly, bone marrow trephine samples were sectioned at 4µm thickness and mounted on SuperFrost slides. Two trephine sections were mounted per slide. Region of Interest (ROI) selection was undertaken in the ACRF translational research laboratory. ROI were selected based on presence of dual CD3/CD45 staining. Six ROIs of 300µm circles were selected. A pre-designed GeoMX™ DSP panel was applied to each region to determine the expression of 57 proteins. The bioinformatics pipeline including data exploration and quality checks, differential expression analyses have been detailed previously and are described further in supplementary information (13).

### Peripheral blood TCR sequencing

Archival DNA from fractionated CD3+ peripheral blood cells remaining post clinical testing from all patients at D30 and D100 post alloSCT was used to perform the analysis as previously described (15).

### Single cell RNA-sequencing and data analysis

scRNA-seq coupled with single-cell T cell receptor/B cell receptor sequencing (scTCR/BCR-seq) analysis for patients and HDs was performed with the 10x Genomics Single Cell Immune Profiling Solution v 2 (Chromium Single Cell 5’ Reagent Kit v2, Cat# 1000263), following the manufacturer’s protocol (www.10xgenomics.com) (16). Library preparation, sequencing, data processing, and quality control were done following previously published pipeline (17). Differentially expressed genes were defined with the FindMarkers function in Seurat. Gene Set Enrichment Analysis (GSEA) was done based on fold changes of all detected genes (http://software.broadinstitute.org/gsea). Comparison between two and more groups was performed using GraphPad Prism v.10.2.1, and results were shown as mean ± standard derivation. Statistical analysis was performed using two-sided unpaired Mann-Whitney test for two groups, and analysis of variance for three or more groups. p < 0.05 was considered statistically significant.

Full details regarding key materials and resources utilized in this manuscript and additional experimental and analytical procedures are described in the Supporting Information.

## Results

### Clinical features of PGF following alloSCT

**Figure 1:**
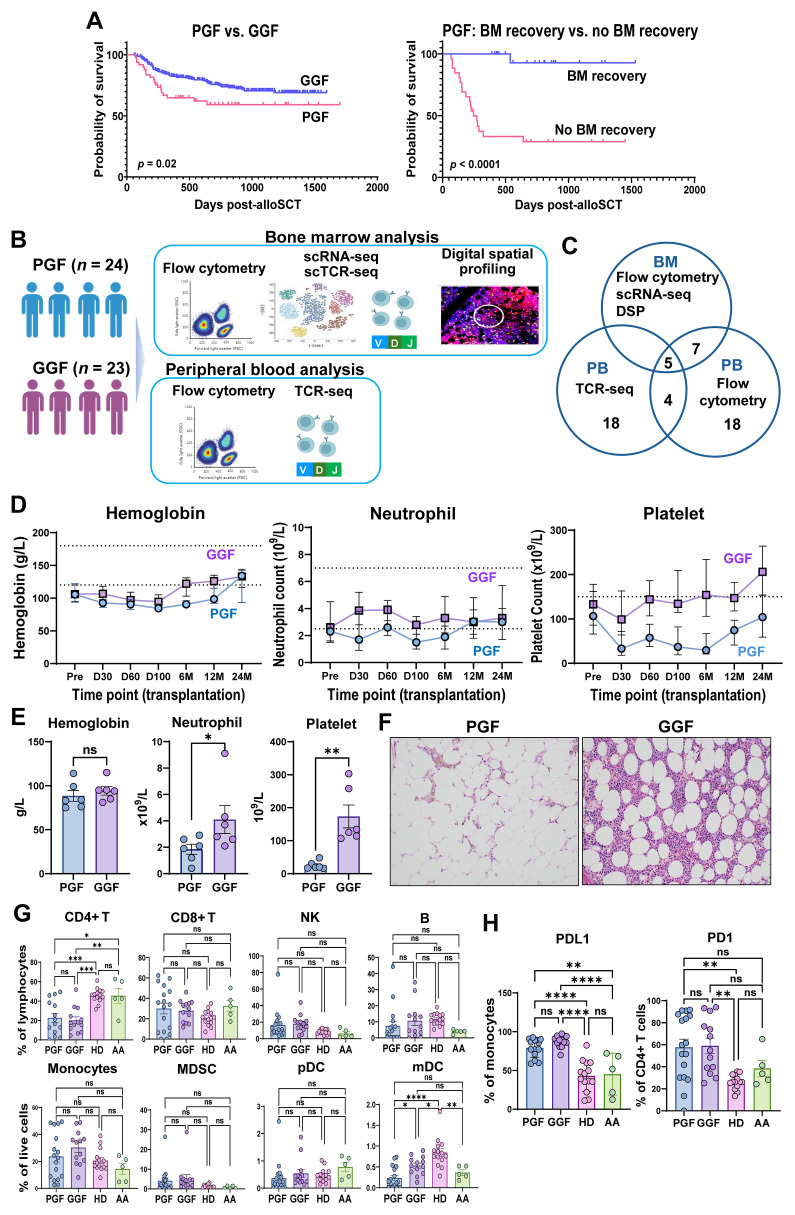
Hematologic features of PGF and immunoprofiles of PB samples. **(A)** Survival of patients with PGF status and duration of PGF. Left panel, survival of PGF (red) vs. GGF (blue); right panel, survival of PGF without BM recovery (red) vs. no BM recovery (blue). **(B)** Experimental workflow. PBMC, BMMNC, and BM biopsy samples from 24 PGF patients, 23 matched GGF patients, and HDs were subjected to multicolor flow cytometry to profile subpopulations of immune cells, hematopoietic cells, and HSPC; to immunoSEQ assay to profile TCR repertoires; to scRNA-seq and scTCR-seq using the 10x Genomics platform; and to DSP of BM tissues. **(C)** Multiple modalities of experiments were done with samples for different subsets of patients. **(D)** Dynamics of blood counts (hemoglobin, neutrophil, and platelet) from pre-transplantation to until 2 years after transplantation. PGF (blue) and GGF (violet) groups are depicted. A dashed line shows a normal reference range for each parameter. **(E)** Blood counts (hemoglobin, neutrophil, and platelet) at time of BM sampling (at day 100 post-transplantation) were compared between PGF (*n* = 6) and GGF (*n* = 4) patients whose BMMNCs were used for scRNA-seq and scTCR-seq. **(F)** Histology images of BM biopsy samples with hematoxylin and eosin staining from representative PGF and GGF patients. **(G)** Flow cytometry was performed to profile major cell populations in peripheral blood. Percentages of CD4^+^ T cells, CD8^+^ T cells, NK cells, B cells, monocytes, MDSC, pDC, and mDC were compared among PGF (*n* = 14), GGF (*n* = 13) patients, HDs (*n* = 14), and patients with AA (*n* = 5). **(H)** Expression of PDL1 on monocytes and expression of PD-1 on T cells were compared among PGF (*n* = 14), GGF (*n* = 13) patients, HDs (*n* = 14), and patients with AA (*n* = 5). Statistical analysis was performed using the two-sided unpaired Mann-Whitney test **(E)** or the ordinary one-way ANOVA test **(G, H)** and these data are shown with mean values ± SEM. *p value < 0.05; **p value < 0.01; ***p value < 0.001; ****p value < 0.0001; ns, no statistical significance. PB, peripheral blood; BM, bone marrow; PGF, poor graft function; GGF, good graft function; HDs, healthy donors; PBMC, peripheral blood mononuclear cell; BMMNC, BM mononuclear cell; HSPC, hematopoietic stem and progenitor cell; TCR, T cell receptor; DSP, digital spatial profiling; SEM, standard error of the mean; NK, natural killer cells; MDSC, myeloid-derived suppressor cells; pDC, plasmacytoid dendritic cells; mDC, myeloid dendritic cells; mDC, myeloid dendritic cells; AA, aplastic anemia.

Our group previously reported the clinical risk factors and outcomes associated with PGF following alloSCT (18). As a follow-up study, we evaluated outcomes in patients with PGF in a cohort of additional 328 patients treated at our center between 2017 and 2020. In this cohort, 49 patients met the criteria for PGF. These patients exhibited significantly worse predicted 2-year overall survival (OS) compared to those without PGF (60% vs. 75%, *p* = 0.022) (**Figure 1A**). Patients who had not achieved BM recovery by their last follow-up demonstrated markedly inferior OS (*p* < 0.0001), with a median OS of 246 days, compared to a median OS that was not reached in those who recovered marrow function (**Figure 1A**). Twenty-three of 49 PGF patients had recovered their BM functions by last follow-up, with a median duration of cytopenias of 142 days (range, 51–789 days). Patients with PGF had worse OS with those without PGF and experience a prolonged duration of cytopenias.

### PGF after hematopoietic cell transplantation mimics AA

A multiomic approach was applied to PB and BM samples from 24 patients with PGF and 23 patients with GGF. The patients were matched for baseline variables including conditioning intensity, donor sources, and CMV serostatus (**Table 1**). Twelve patients had their BM evaluated by flow cytometry and scRNA-seq, while corresponding trephine biopsies were assessed by spatial proteomics. All these BM samples were collected at day100 post-stem cell infusion. Both PGF and GGF patients had similar CD3 negative (median 100% for both groups) and CD3 positive (99% versus 98%) chimerism at D100 (**Table 1**). PB samples were evaluated by flow cytometry and bulk RNA-seq to assess TCR profiles in selected CD3^+^ cells (**Figures 1B, C**).

**Table 1:** Clinical characteristics of patients.

Variable	PGF (*n* = 24)	GGF (*n* = 23)	*p*-value
Age	Median: 60 IQR (54.50-64.0)	Median: 58 IQR (51.75-62.0)	0.27
Disease			
AML	11	10	N/A
MDS	3	4	
NHL	6	5	
MDS/MPN overlap	2	0	
MPN	2	1	
Donor type			
MSD	9	8	0.95
Haploidentical	2	2	
MUD	13	10	
Intensity			
MAC	2	2	1.00
RIC	22	18	
CMV match			
D-R-	3	4	0.46
D-R+	4	4	
D+R-	7	2	
D+R+	10	10	
Viral reactivation post-alloSCT	12	11	0.88
GVHD post-alloSCT	8	1	0.02*
D100 CD3 neg Chimerism	Median: 100 IQR (98.75-100)	Median: 100 IQR (99-100)	0.53
CD100 CD3 pos Chimerism	Median: 99 IQR (97-100)	Median: 98 IQR (93-100)	0.11

AML, acute myeloid leukemia; MDS, myelodysplastic syndromes; NHL, non-Hodgkin lymphoma; MPN, myeloproliferative neoplasms; MSD, multiple sulfatase deficiency; MUD, mud disease; MAC, myeloablative conditioning; RIC, reduced intensity conditioning; CMV, cytomegalovirus; D-R-, donor negative and recipient negative; D-R+, donor negative; D+R-, donor positive and recipient negative; D+R+, donor positive and recipient positive; SCT, stem cell transplantation; GVHD, graft versus host disease; PGF, poor graft function; GGF, good graft function; IQR, interquartile range. *P value < 0.05.

As expected, PGF patients had lower hemoglobin, neutrophil, and platelet counts compared to GGF patients (**Figure 1D**). Among subsets included in BM analysis, blood counts at day100 revealed significantly reduced neutrophil (p=0.04) and platelet counts (p=0.0017) in PGF (**Figure 1E**). BM cellularity was also markedly lower in patients with PGF compared to those with GGF (**Figure 1F**).

In PB, relative proportions of CD4^+^ T cells, CD8^+^ T cells, natural killer (NK) cells, B cells, monocytes, myeloid-derived suppressor cells (MDSC), plasma dendritic cells (pDC), and myeloid dendritic cells (mDC) were similar between PGF and GGF (**Figure 1G**). Interestingly, despite most GGF patients receiving BM from HLA-matched donors and exhibiting low frequency of GVHD and viral reactivation (**Table 1**), expression levels of PD1 on CD4^+^ T cells (p=0.0010) and PDL1 on monocytes were significantly higher (p<0.0001) compared to those in HDs (**Figure 1H**), and comparable to levels observed in PGF patients.

### Immunoprofiling of BM in alloSCT patients

**Figure 2:**
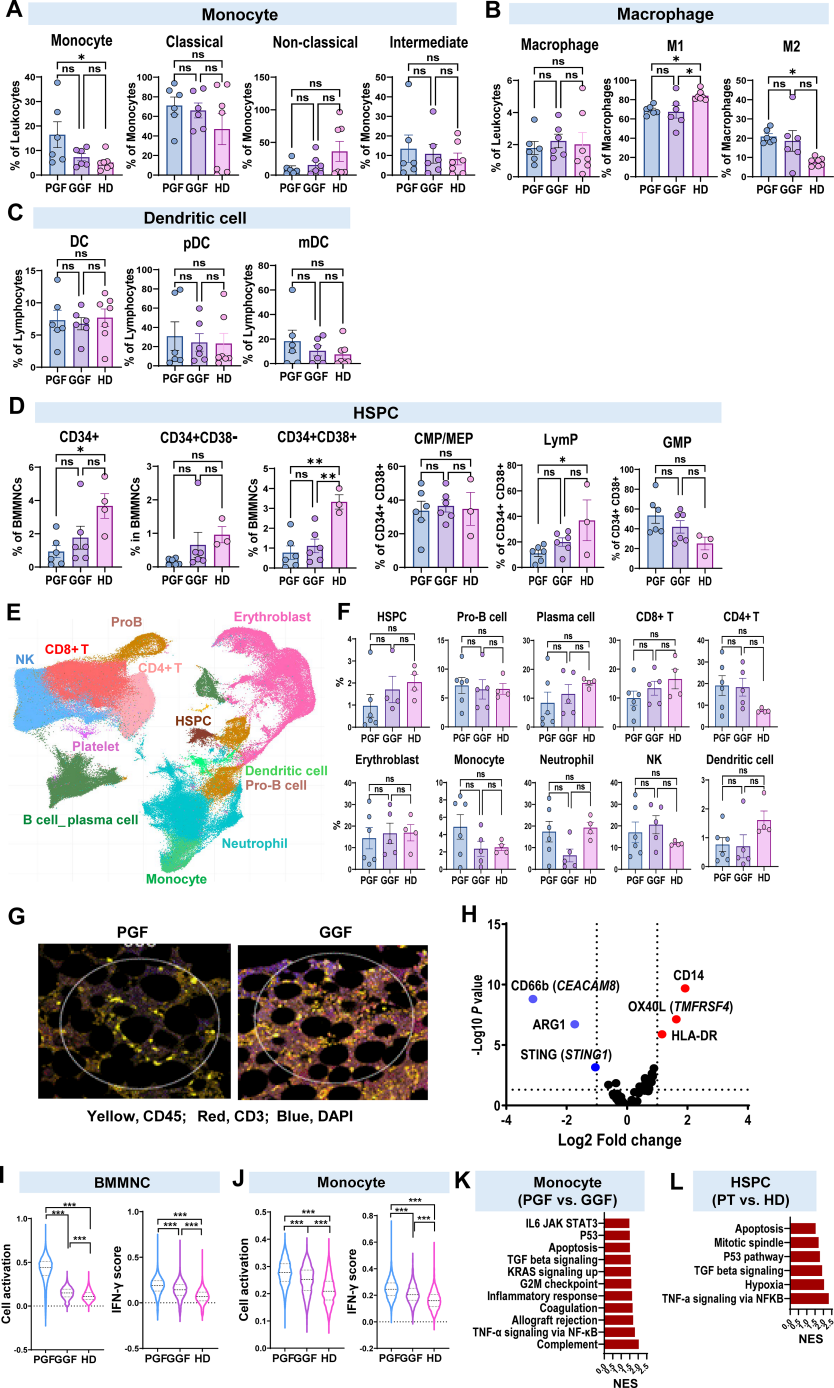
hematopoietic and immune cell populations of BM profiled with flow cytometry, scRNA-seq and DSP. Flow cytometry was performed to profile major cell populations in BM. Percentages of monocyte **(A)**, macrophage **(B)**, dendritic cell **(C)**, HSPC **(D)**, and their subpopulations were compared among PGF (*n* = 6), GGF (*n* = 6) patients, and HDs (n = 7). **(E)** A UMAP plot of single-cell gene expression in BMMNCs from PGF (*n* = 6), GGF (*n* = 5) patients, and HDs (*n* = 4). Cells are colored by types. **(F)** Bar charts show percentages of these cell populations in individual patients and HDs. **(G)** A top panel shows an example of an immunofluorescence image prior to DSP demonstrates regions of interest selected across a sample. Bottom three panels represent examples of cellularity in patients with PGF or GGF, and HDs, respectively, showing CD45 (Green) and CD3 expression (red). **(H)** A volcano plot of multiple protein levels in BM tissues of PGF compared to GGF profiled by DSP. Upregulated and downregulated proteins in PGF are indicated in red and blue, respectively. **(I)** Comparison of relative inflammatory pathway scores (cell activation and IFN-γ signaling scores) of BMMNCs evaluated by scRNA-seq in PGF (*n* = 6), GGF (*n* = 5), and HDs (*n* = 4). **(J)** Comparison of relative inflammatory pathway (cell activation and IFN-γ signaling) scores of monocytes evaluated by scRNA-seq in PGF, GGF patients, and HDs. *p* values with the ordinary one-way ANOVA test are shown. **(K)** GSEA of genes expressed in monocytes from PGF, GGF patients, and HDs. Normalized enrichment scores for the GSEA pathways are plotted, showing higher enrichment of the inflammatory pathways in monocytes. **(L)** GSEA of genes expressed in HSPCs of PGF, GGF patients, and HDs. Normalized enrichment scores for the GSEA pathways are plotted, showing enrichment of cell cycling and cell apoptosis genes, and immune related genes in HSPC in post alloSCT. Statistical analysis was performed using the ordinary one-way ANOVA test **(A-D, F)** and data are shown with mean values ± SEM. *p value < 0.05; **p value < 0.01; ***p value < 0.001; ****p value < 0.0001; ns, no statistical significance. M1, M1 macrophage; M2, M2 macrophage; DC, dendritic cell; CMP, multipotent common myeloid progenitor; MEP, megakaryocyte-erythroid progenitor; LymP, lymphoid progenitors; GMP, granulocyte-monocyte progenitors. UMAP, Uniform Manifold Approximation and Projection; GSEA, Gene Set Enrichment Analysis; NES, normalized enrichment score; alloSCT, allogeneic stem cell transplantation.

To understand changes following alloSCT and evaluate potential differences between PGF and GGF patients, we profiled hematopoietic and immune cell populations in BM. Within myeloid populations, there was a general trend toward increased intermediate monocytes and decreased non-classical monocytes post-alloSCT. Additionally, PGF patients exhibited higher levels of total monocytes (p= 0.045) and M2 macrophages than HDs (p= 0.0078) (**Figures 2A-C**; **Supplementary Figure 1**).

At day100 post-alloSCT, patients exhibited lower levels of CD34^+^ hematopoietic stem and progenitor cells (HSPCs) compared to HDs, with PGF patients exhibiting significantly lower CD34+ HSPCs (p= 0.022) (**Figure 2D**), consistent with reduced HSC numbers following alloSCT. Additionally, there was a trend toward a higher proportion of granulocyte-monocyte progenitors (GMP) and a lower proportion of lymphoid progenitors (LymP) in alloSCT patients, suggesting skewed differentiation favoring the myeloid lineage.

### Transcription profiling of BM cells in PGF and GGF patients

To investigate cellular and transcriptomic changes of BM cells following transplantation, we performed scRNA-seq on BMMNCs from post-transplantation patients (6 PGF and 5 GGF). Based on established marker genes (19), BMMNCs were annotated to major cell populations, including HSPCs, erythroblasts, dendritic cells, neutrophils (across all differentiation stages), monocytes (across all monocytic lineage differentiation stages), ProB, B cell/plasma cells, CD8^+^ T, CD4^+^ T cells, and NK cells (**Figures 2E**; **Supplementary Figure 2A**). Post-transplantation patients tended to have lower HSPCs compared with HDs, with lower numbers in PGF than in GGF patients, consistent with flow cytometry results. Additionally, post-allSCT patients tended to have elevated proportions of CD4^+^ T cells and NK cells than HDs, while PGF patients tended to have an increased frequency of monocytic lineage than both GGF patients and HDs (**Figure 2F**).

Digital spatial profiling (DSP) of BM trephine biopsies from PGF and GGF patients revealed a decreased CD45^+^ cell density in PGF patients than in GGF patients (**Figure 2G**), consistent reduced hematopoietic cells in PGF patients. Additionally, PGF patients showed significantly higher expression of the monocytic marker CD14, HLA-DR, and the costimulatory molecule OX40L compared to GGF patients (**Figure 2H**).

We compared gene expression profiles of total BMMNCs among PGF, GGF patients, and HDs, and found elevated expression of genes related to cell activation and IFN-γ signaling in post-alloSCT patients, particularly in PGF patients (**Figures 2I, J**). Monocytes from PGF patients exhibited higher expression of genes associated with cell activation and multiple inflammatory pathways, including IFN-γ signaling, TNF-α via NF-κB, allograft rejection, inflammatory response, and IL6 JAK STAT3 signaling, as well as the G2M checkpoint cell cycling pathway, TGF beta signaling, and the apoptosis pathway (**Figure 2K**). These findings indicate enhanced monocyte proliferation and activation, compensatory immune regulation, and subsequent apoptosis. In addition, HSPCs from post-transplantation patients exhibited upregulation of the immune and cellular stress pathways such as TNFα via NFκB, hypoxia, and P53 signaling, as well as cell apoptosis (**Figure 2L**).

### T cells are highly clonally expanded and display activated phenotypes post-transplantation

**Figure 3:**
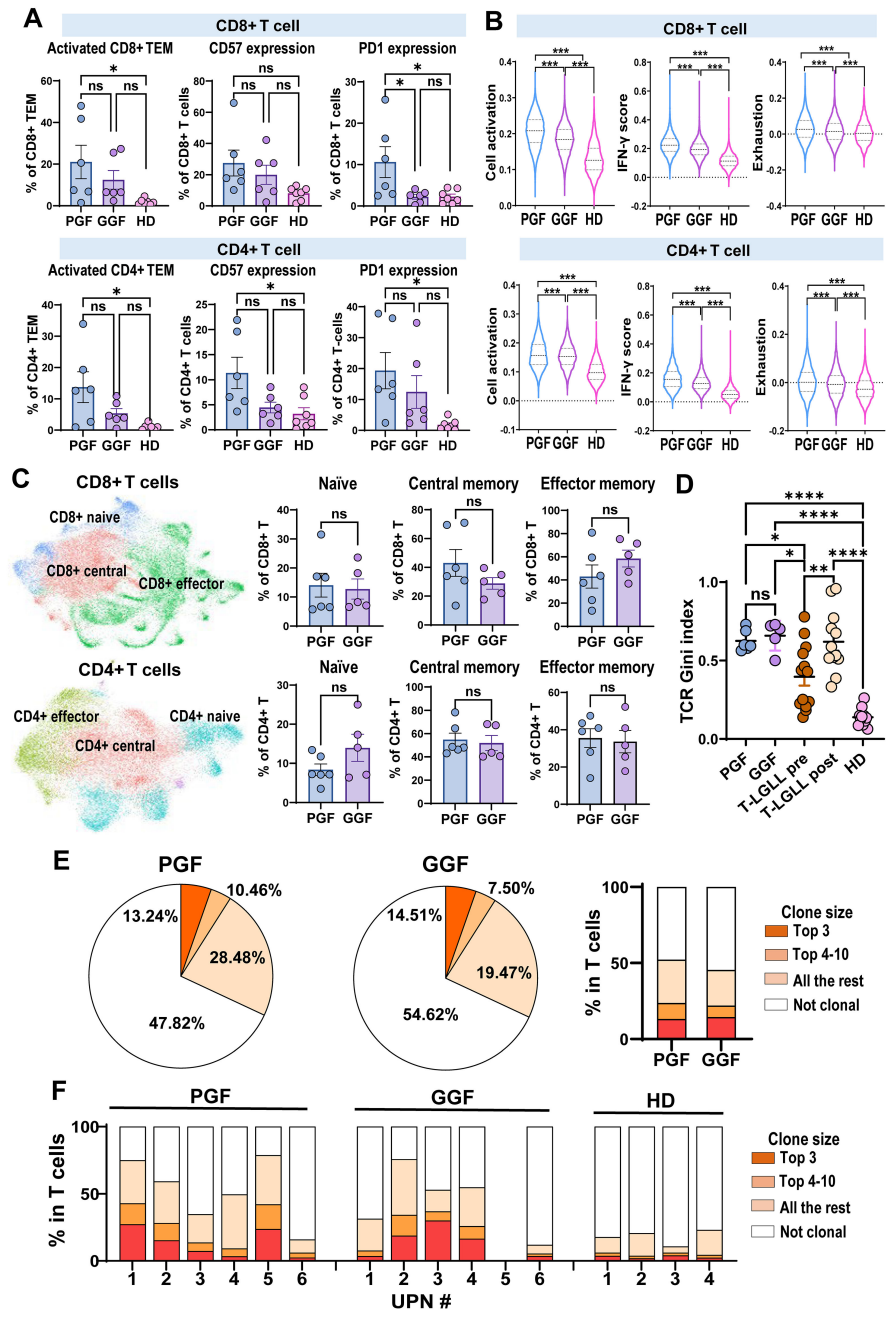
T cells are activated and clonally expanded in PGF and GGF. **(A)** Flow cytometry was performed to profile T cells in BM. Percentages of activated TEM, CD57 expression, and PD1 expression in CD4^+^ T and CD8^+^ T cells were compared among PGF (*n* = 6), GGF (*n* = 6) patients, and HDs (*n* = 7). **(B)** Comparison of relative inflammatory pathway (cell activation, IFN-γ signaling, and exhaustion) scores of CD4^+^ T and CD8^+^ T cells evaluated by scRNA-seq in PGF (*n* = 6), GGF (*n* = 5), and HDs (*n* = 4). **(C)** A UMAP plot of single-cell gene expression in CD8^+^ T (top panel) and CD4^+^ T (bottom panel) cells of PGF (*n* = 6), GGF (*n* = 5) patients, and HDs (*n* = 4). Cells are colored by types. Percentages of subpopulations in CD4^+^ T and CD8^+^ T cells were compared among PGF (*n* = 6), GGF (*n* = 5) patients, and HDs (n = 4). **(D)** Gini index of TCR clonality in T cells in BM of PGF (*n* = 6) and GGF (*n* = 5). Gini index of patients with T-LGLL (pre- and post-treatment) and HDs included in a previously published dataset was also included (33). **(E)** Pie charts summarizing frequency of T cell clones in PGF (*n* = 6) and GGF (*n* = 5) patients. A bar chart showing frequency of T cell clones (% in T cells) with clone sizes of top 3, top 4-10, all the rest clones (with at least 2 cells with identical TCR), and cells without clonal TCR in samples. **(F)** A bar chart showing frequency of T cell clones (% in T cells) with clone sizes of top 3, top 4-10, all the rest clones (with at least 2 cells with identical TCR), and cells without clonal TCR in individual samples. Statistical analysis was performed using the ordinary one-way ANOVA test **(A, B, D)** or the two-sided unpaired Mann-Whitney test **(C)**. Data in **(A, C)** are shown with mean values ± SEM. *p value < 0.05; **p value < 0.01; ***p value < 0.001; ****p value < 0.0001; ns, no statistical significance. TEM, terminal effector memory T cells; T-LGLL, T-cell large granular lymphocytic leukemia.

We next profiled subpopulations and functional surface markers of CD4^+^ T and CD8^+^ T cells using flow cytometry. Patients with PGF exhibited a higher percentage of activated CD4^+^ TEMs (p=0.005) and CD8^+^ T cells TEMs (p= 0.015), (**Figure 3A**) compared to HD and higher expression of PD1 compared to both HD and GGF patients particularly with regards to CD8 T-cells (p= 0.019). These data suggest that T cells in PGF patients adopt an activated yet functionally exhausted phenotype.

Functional gene expression scores were assessed using scRNA-seq analysis, including cell activation, IFN-y signaling, and exhaustion scores. Post-alloSCT patients exhibited significantly elevated scores across all three categories compared to HDs, with PGF patients showing the highest scores (**Figure 3B**), aligning with flow cytometry findings. Subsets analysis of CD4^+^ T and CD8^+^ T cells including naïve, central memory, and effector memory revealed no significant differences between PGF and GGF patients (**Figure 3C**).

We then assessed T cell clonal expansion at a single cell level; a clone was defined when at least two T cells exhibited identical TCR sequences. Overall, PGF patients exhibited greater T cell clonal expansion in BM compared to GGF patients (**Figure 3E**). However, the degree of clonal expansion varied among individuals. Both PGF and GGF patients demonstrated higher TCR usage and reduced TCR diversity than HDs (**Figures 3D, F**). These data indicate that, at 100 days post-transplantation, T cells are still undergoing repertoire reconstitution, with limited TCR diversity, and this change is more prominent in PGF than in GGF patients.

### Clonally expanded T cells exhibit activated phenotypes post-alloSCT, with distinct clonal dynamics of TCR usage in PGF and GGF patients

**Figure 4:**
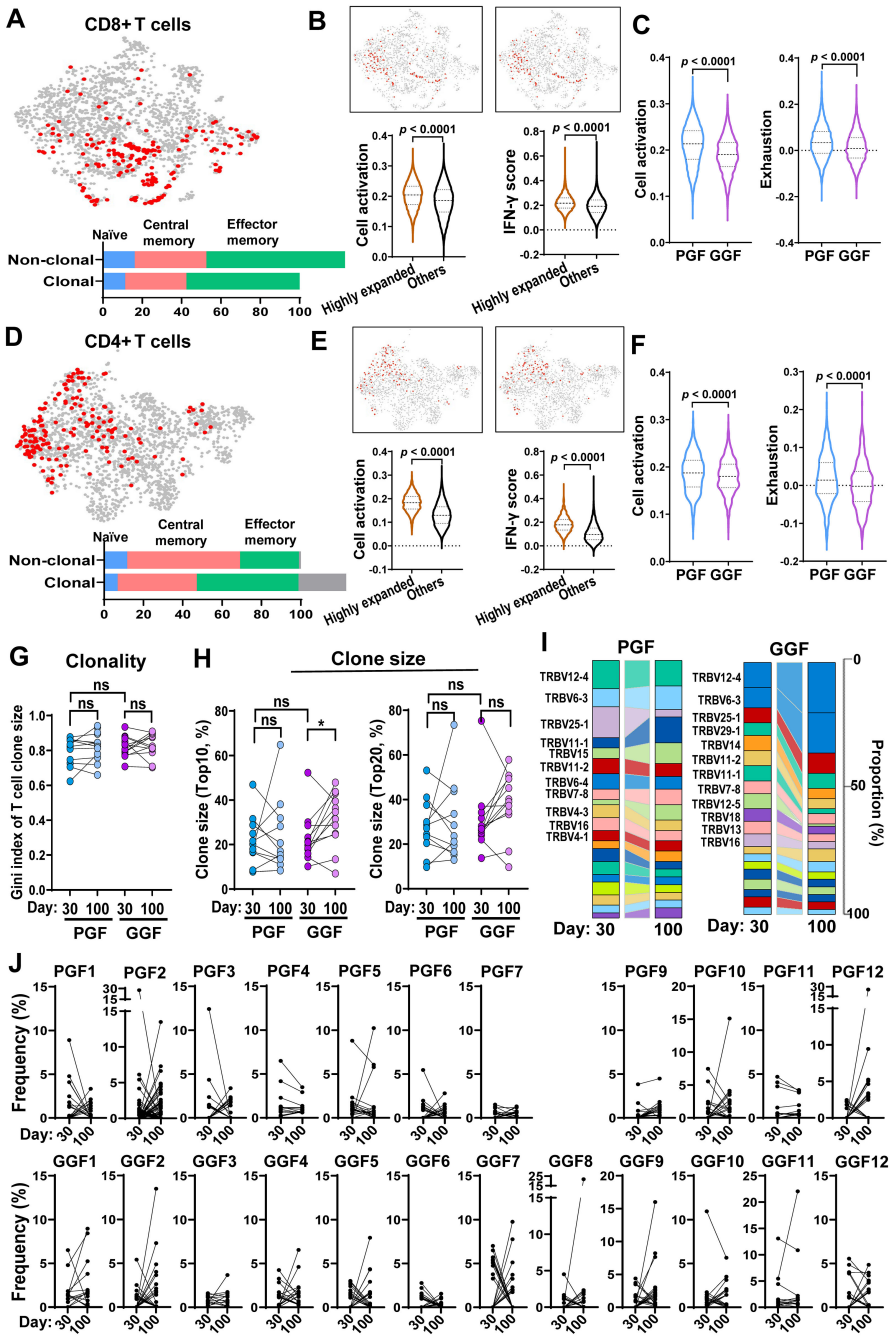
Activation features of T cells and clonal dynamics in PGF and GGF post-transplantation. **(A)** In a UMAP only showing CD8^+^ T cells, T cell clones ranking top 5% are highlighted in red. Percentages of naïve, central memory, and effector memory subtypes were categorized in clonally expanded (with at least 2 cells with an identical TCR) and non-clonally expanded CD8^+^ T cells. **(B)** In CD8^+^ T cells, top 10% cells expressing the highest cell activation score and IFN-γ signaling score are highlighted in red and all the rest in grey. In CD8^+^ T cells, expression of cell activation genes and IFN-γ genes were plotted for clonally highly expanded T cells (clone size ≥ 10) and non-clonally expanded T cells. **(C)** In highly clonal expanded CD8^+^ T cells, expression of cell activation genes and exhaustion genes were plotted for PGF and GGF samples. **(D)** In a UMAP only showing CD4^+^ T cells, T cell clones ranking top 5% are highlighted in red. Percentages of naïve, central memory, and effector memory subtypes were categorized in clonally expanded (with at least 2 cells with an identical TCR) and non-clonally expanded CD4^+^ T cells. **(E)** In CD4^+^ T cells, top 10% cells expressing the highest cell activation score and IFN-γ signaling score are highlighted in red and all the rest in grey. In CD4^+^ T cells, expression of cell activation genes and IFN-γ genes were plotted for clonally highly expanded T cells (clone size ≥ 10) and non-clonally expanded T cells. **(F)** In highly clonally expanded CD4^+^ T cells, expression of cell activation genes and exhaustion genes were plotted for PGF and GGF samples. **(G)** With ImmunoSEQ data of peripheral blood samples, Gini index of T cell clone sizes were plotted for PGF and GGF patients at day 30 and day 100 post-transplantation. **(H)** With ImmunoSEQ data of peripheral blood samples, T clone sizes of top10 (left) and top 20 (right) were plotted for PGF and GGF patients at day 30 and day 100 post-transplantation. **(I)** Flow diagrams showing T cell clonotypes (defined as TRBV gene usage and CDR3β sequence) between samples of day 30 and day 100. Left and right panels show grouped TCRs from patients with PGF and GGF, respectively. **(J)** Clone size dynamics. For each individual, a scatter plot represents top10 and top 20 clone sizes at day 30 and day 100 post-transplantation, respectively. Statistical analysis was performed using the two-sided unpaired and paired Mann-Whitney test **(B, C, E–H)**. *p* < 0.0001; ns, no statistical significance.

To examine T cell clonality and corresponding functional changes, we integrated scRNA-seq and scTCR-seq data to link TCR sequences with gene expression profiles. In both CD4^+^ T and CD8^+^ T cells, clonally expanded T cells were predominantly enriched in the effector memory subtypes. Highly expanded T cell clones (defined as clones comprising at least 10 cells sharing identical TCR sequences) displayed elevated cell activation and IFN-γ signaling scores than the rest of T cells (**Figures 4A, B, D, E**; **Supplementary Figure 3**). Notably, clonally expanded T cells in PGF patients exhibited even higher cell activation and exhaustion scores than those in GGF patients (**Figures 4C, F**). These data indicate clonally expanded T cells are broadly activated and pro-inflammatory following alloSCT, with the phenotypes being more pronounced in PGF than in GGF.

We further assessed TCR repertoires using ImmunoSeq in 26 patients (13 PGF and 13 GGF) at both day 30 and day 100 post-transplantation. T cell clonality remained persistently high at both timepoints, indicating sustained oligoclonality during the early post-alloSCT period (**Figure 4G**). Clones were primarily shared between different timepoints of the same individual, but not sharing across different individuals, indicating lack of a common T cell immunogenetic signature for either PGF or GGF (**Supplementary Figures 4**, **5**).

We examined clone sizes and dynamics post-alloSCT. In GGF, both top 10 and top 20 clones tended to increase in size from day 30 to day 100, whereas PGF patients showed variable clonal expansion patterns (**Figure 4H**). Top TCRVB usage remained equivalent at day30 and day100, and were shared across PGF and GGF patients (**Figure 4I**). Specifically, TRBV11–1 and TRBV15 clone sizes expanded over time in PGF patients, while TRBV12-4, TRBV6-3, and TRBV25–1 clone sizes expanded in GGF patients. Most PGF and GGF patients exhibited dynamic clonal patterns characterized by contraction of pre-existing clones and emergence of new clones (**Figure 4J**), indicating that while oligoclonal T cell expansion persists at both timepoints, substantial clonal drifting occurs during the early post-transplantation period.

### Immune cell interactions contribute to a hyper-inflamed BM microenvironment

**Figure 5:**
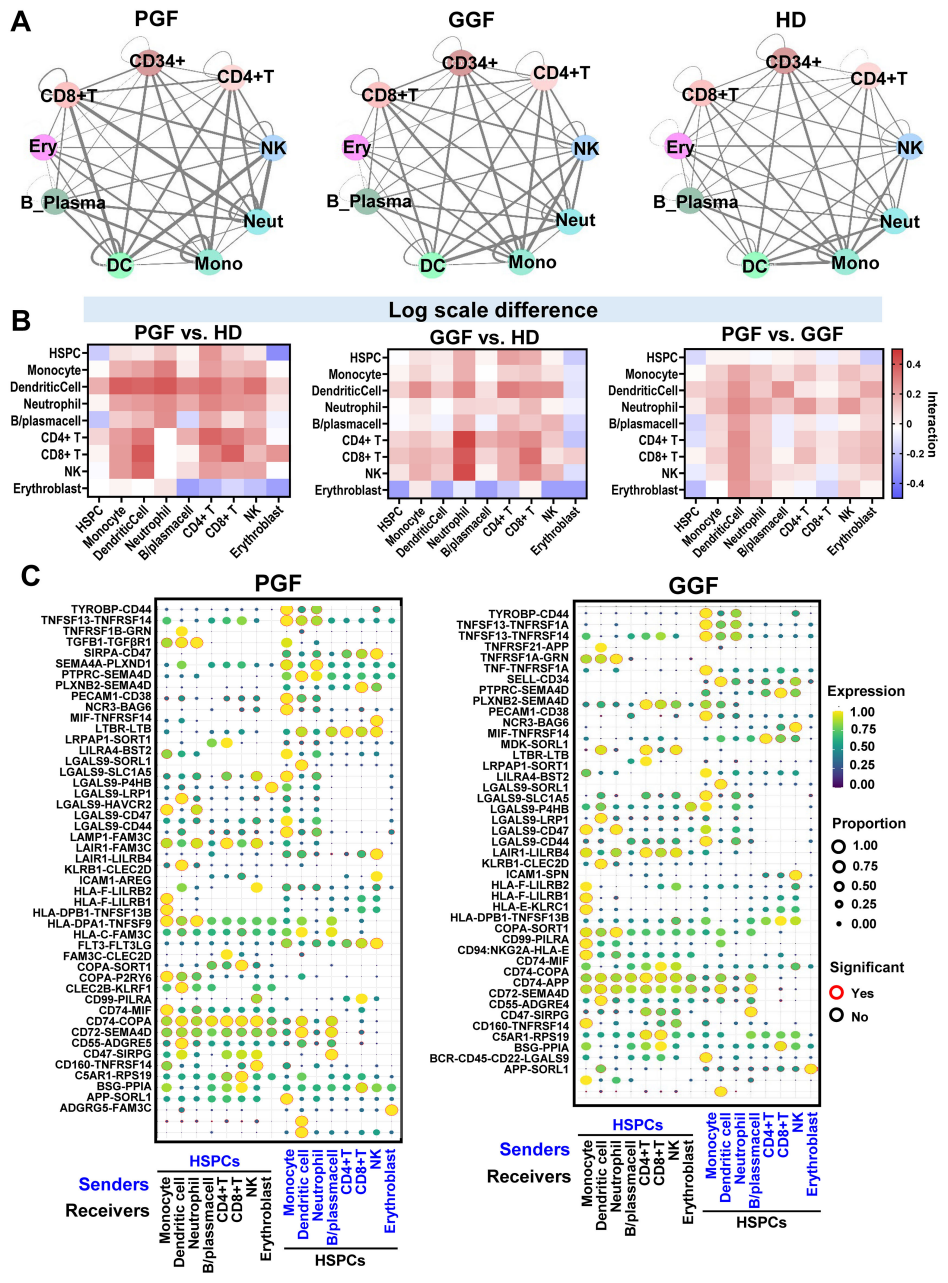
Cell-cell interactions between immune cells and hematopoietic cells are enhanced post-transplantation. **(A)** Ligand-receptor pairs among cell types in BMMNCs were estimated by CellPhoneDB. Color legends for cell types are the same as in **Figure 3A**. Thickness of lines connecting cell types indicates a total number of ligand-receptor pairs between two cell types estimated by CellPhoneDB. In general, there were more ligand-receptor interactions among cell types in BMMNCs of PGF (left) and GGF (middle) patients than in those of HDs (right). **(B)** Heatmaps showing LogFC of cell-cell interaction scores estimated by CellPhoneDB across cell types in BMMNCs in PGF patients relative to HDs (left), GGF patients relative to HDs (middle), and PGF relative to GGF patients (right). **(C)** Ligand-receptor pairs that were overrepresented in PGF and GGF patients than in HDs across cell types in the BMMNCs were presented with HSPCs as senders (left) and receivers (right). Significance indicates if a ligand-receptor pair is over-represented in pre-treatment samples of patients compared with HDs. B_Plasma, B cell_Plasma cell; Ery, erythroblast; Neut, neutrophil; Mono, monocyte.

To investigate intercellular communications, we used CellphoneDB (20) to computationally infer ligand-receptor interactions across different cell types. Overall cell-cell interactions were enhanced in post-alloSCT patients compared to HDs, and were particularly elevated in PGF patients (**Figure 5A**). Notably, stronger interactions were observed in PGF than in GGF patients among monocytes, HSPCs, T cells, and HSPCs, as well as among various immune cell subsets (**Figure 5B**). These data suggest that cell-cell interactions across cell types in BM are enhanced after transplantation, potentially primed to a hyper-inflamed microenvironment, which may contribute to suppression of HSPC functions under certain conditions. Top ligand-receptor pairs identified between HSPCs and other cell types included TNFSF13-TNFRSF14 and TGFB1-TGFBR1, both of which are associated with immune regulation and cell differentiation; LGALS isoforms interacting with various receptors involved in immune homeostasis; and FLT3-FLT3LG, which plays a role in the regulation of hematopoietic and lymphoid progenitors (**Figure 5C**).

**Figure 6:**
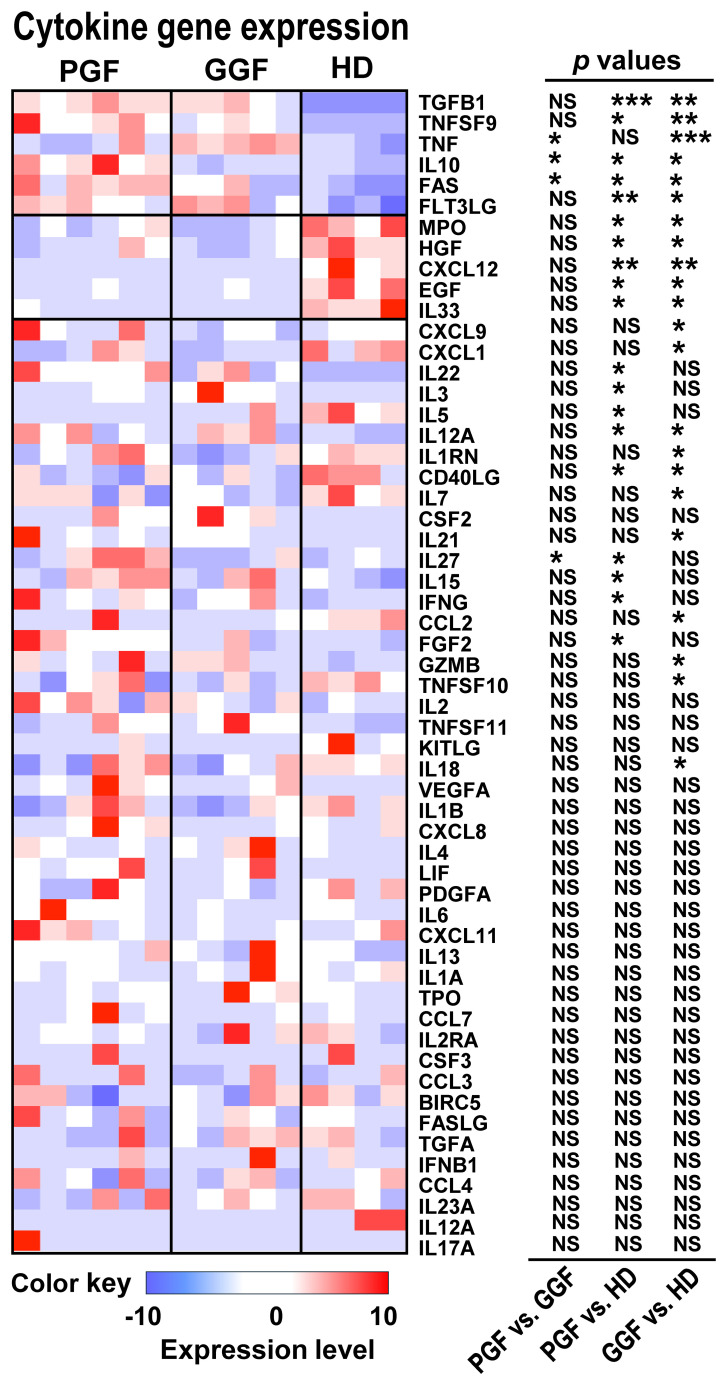
Inflamed BM post-transplantation. Heatmaps show cytokine gene expression levels in BMMNCs of PGF (*n* = 6), GGF (*n* = 5) patients, and HDs (*n* = 4). Three columns on the right indicate *p* values by comparing PGF vs. GGF patients, PGF patients vs. HDs, and GGF patients vs. HDs. The two-sided paired t-test. *p value < 0.05; **p value < 0.01; ***p value < 0.001; ****p value < 0.0001; NS, no statistical significance.

We analyzed cytokine gene expression in BM cells from post-transplantation patients and HDs (**Figure 6**). *TGFB1* and *IL-10* levels were elevated in both PGF and GGF patients compared to HDs, reflecting a compensatory immunoregulatory response. This response was more pronounced in PGF patients, as *IL-10* expression was higher in PGF than in GGF patients. Pro-apoptotic genes *FAS*, *TNF*, and *TNFSF9* were also upregulated in both PGF and GGF patients, suggesting ongoing cellular apoptosis post-alloSCT, regardless of graft function. Notably, *FAS* and *TNF* levels were higher in PGF than in GGF, consistent with features of a late-stage of apoptosis, whereas *TNFSF9/TNF* expression in GGF may reflect early stages of apoptotic activity. Conversely, genes involved in supporting hematopoiesis and the marrow niche such as *MPO*, *HGF*, *CXCL12*, *EGF*, and *IL33* were downregulated in both PGF and GGF patients, suggesting impaired hematopoiesis and a disrupted microenvironment. Furthermore, a subset of pro-inflammatory cytokines, including *IL-27*, *IL-15*, *FGF2*, *IL-22*, and *IFNG*, were predominantly elevated in PGF patients compared to HDs, indicating a heightened inflammatory state in these patients.

## Discussion

Hematopoietic and immune reconstitution following alloSCT is a prolonged and dynamic process, in which the interactions of immune cells and HSCs play important roles in effective hematopoiesis. Understanding the underlying mechanisms of PGF is essential, as patients affected by PGF have limited treatment options and a poor prognosis (18). We previously conceptualized the development of PGF as interplays among seed (HSC), soil (stroma), and climate (immune cell) (21). With the seed, soil, and climate model in mind, we performed the first comprehensive cellular and molecular evaluation of PGF following alloSCT, offering new insights into post-alloSCT hematopoiesis and immune reconstitution. Our current work demonstrate evidence of dysfunctional immune “climate” in post-alloSCT setting, particularly in PGF, which likely impairs the functionality of HSCs.

Regarding a role of the “seed” in our PGF model, our work added to previous studies demonstrating quantitative and qualitative dysfunctions of HSCs post-alloSCT, particularly in patients with PGF. We observed a reduction in HSC numbers, accompanied by upregulation of the apoptotic pathways post-alloSCT. These findings were consistent with a prior study demonstrating *ex vivo* HSCs from patients with PGF exhibit increased levels of reactive oxygen species, DNA damage, and increased expression of apoptotic proteins (22). Interestingly, this study also showed that cryopreserved CD34^+^ donor cells from individuals whose recipients later developed PGF retained normal repopulating capacity when xenografted into mice, highlighting that HSC dysfunction is acquired within the recipient during alloSCT process, rather than being an intrinsic defect of the donor cells.

The dysfunctional immune environment following alloSCT likely contributes to the HSC dysfunction and the development of PGF. Our data highlight numerous significant interactions between HSCs and immune cells in the context of normal hematopoiesis, which appear to be exaggerated and dysregulated in PGF. We imputed that several potentially enhanced ligand-receptor interactions that may mediate immune-driven suppression of HSCs, including TNFSF13-TNFRSF14 and TGF-beta/TGFBR. The TNFSF13-TNFRSF14 interaction, for example, may promote cellular apoptosis when TNFSF13 binds to TNFRS14, a mechanism that could be relevant to the HSC dysfunction in PGF (23, 24). However, a specific role of this particular ligand-receptor interaction in hematopoiesis remains extensively evaluated. In contrast, TGF-beta/TGFBR interactions in HSCs have been studied more extensively; *ex vivo* experiments have shown that TGF-beta can slow down HSC cell cycle progression and suppress self-renewal, potentially contributing to HSC exhaustion and BM failure characteristics of PGF (25). At the cellular levels, numerous interactions between HSCs and immune cells have been characterized in experimental models. Activated CD8^+^ T cells promote HSC differentiation and inhibit self-renewal capacity. Conversely, memory CD8^+^ T cells support HSC self-renewal and contribute to HSC maintenance and recovery (26). Similarly, monocyte-derived macrophages control many aspects of HSC biology, including mobilization, engraftment, and hematopoiesis (27). The activation and clonal expansion of CD8^+^ effector memory T cells, along with increased macrophage/monocyte activation, may disrupt these supportive roles in hematopoiesis and instead contribute to suppression of the HSC function in PGF. Together, these ligand-receptor and immune cell interactions suggest mechanisms of HSC apoptosis and exhaustion in PGF and warrant further assessment in mechanistic studies.

We observed several notable changes in the T-cell compartment following alloSCT. TCR repertoire restriction is a known occurrence post-alloSCT, representing selective mobilization of donor-derived T cell clones and selection pressures in the recipient environment, such as CMV reactivation (28–30). Consistent with prior studies, TCR restriction appears to be accompanied by clonal expansion, primarily of CD4^+^ and CD8^+^ effector memory T cells (29). Interestingly, we observed that clonally expanded T-cells in PGF exhibited increased activation. This provides a molecular basis for the clinical observation that PGF is associated with aberrant expansions of T helper 1 and cytotoxic T cells (10, 12).

Concurrent to T cell activation, we also observed prominent activation of monocytes following alloSCT, particularly in patients with PGF. A recent publication, utilizing CyTOF to evaluate post-alloSCT myelopoiesis, has demonstrated that innate immune cells, including monocytes and dendritic cells, exhibit upregulation of the inflammatory response pathways, such as IL-Stat5, IFN-alpha, and IFN-gamma (4). Our data suggest that the upregulation of these pathways is even more pronounced in PGF compared to GGF, further supporting the presence of an exaggerated innate immune response in PGF.

Our data clearly demonstrate that the post-alloSCT immune environment is markedly distinct from normal, characterized by features such as T-cell clonal restriction upregulation of exhaustion molecules such as PD1 and PDL1. This altered immune state may create a primed condition for the development of PGF. Clinically, PGF is associated with several inflammatory complications following alloSCT, including early ICU admission, GVHD, and viral reactivation (21). These events may act as triggers within an already primed immune landscape, promoting immune-mediated HSC suppression and ultimately contributing to the onset of PGF.

Patients with PGF experience poorer OS and prolonged cytopenias, which are resource-intensive to manage due to increased healthcare utilization and transfusion requirements (31). These factors also likely have impacts on patient quality of life. Currently, there are limited effective treatment options for PGF, highlighting the urgent need for novel therapeutic approaches. This work provides an initial evidence base for potential usage of immune modulatory therapies in the management of PGF, an area that remains largely unexplored. To date, most reported interventions have focused on enhancing stem cell functions with agents such as eltrombopag or CD34^+^ stem cell boost (21, 32). Immune modulation may serve as a valuable adjunct to these strategies, and its therapeutic potential warrants further investigation through interventional clinical studies.

Our work is limited by a small patient cohort and a lack of in-depth mechanistic investigations. Additionally, we did not evaluate a potential role of stromal cells in the development of PGF, which has been highlighted as a contributing factor by other groups (33–35). Future studies incorporating larger patient numbers and functional assays, including stromal-immune interactions, and in specific patient cohorts ie. T cell depleted alloSCT and haploidentical cohort, will be important to fully elucidate the multifactorial nature of PGF in different settings.

## Data Availability

The datasets presented in this study can be found in online repositories. The names of the repository/repositories and accession number(s) can be found in the article/**Supplementary Material**.
