# Supplementary material for: T cell and monocyte activation in concert with hematopoietic stem cell interactions shapes the post-allogeneic transplant immune landscape in poor graft function
Source: Front Immunol. 2026 Feb 4;17:1750093. doi: 10.3389/fimmu.2026.1750093 (PMC12913120; doi:10.3389/fimmu.2026.1750093)
Supplement: Supplementary file 1 [file DataSheet1.pdf]

Supplementary Figures

T cell and monocyte activation in concert with hematopoietic stem cell interactions shapes the post-allogeneic transplant immune landscape in poor graft function

Short title: multiomic evaluation of hematopoiesis and immune landscape post-allogeneic stem cell transplantation

Keywords: hematopoietic cell transplantation; single-cell RNA sequence; T cell receptor; immune cell interactions; immune reconstitution

Ashvind Prabahran,[1,2,3,4,*] Zhijie Wu,[4,*] Shouguo Gao,[4] Huw Morgan,[2] Nicholas Holzwart,[2] Mandy Ludford-Menting,[2] Mayani Rawicki,[2] Jessica Klass[1,2] Ray-Mun Koo,[1,2,3] Clarissa Wilson,[5] Piers Blombery,[5] Chin Wee Tan,[6] Saanvi Indukuri,[4] Lynette Chee,[1,2,3] David Ritchie,[1,2,3] Neal S. Young,[4] Xingmin Feng,[4,†] and Rachel Koldej,[2,3,†]

*A.P. and Z.W. contributed equally to this work.

[†]co-last authors

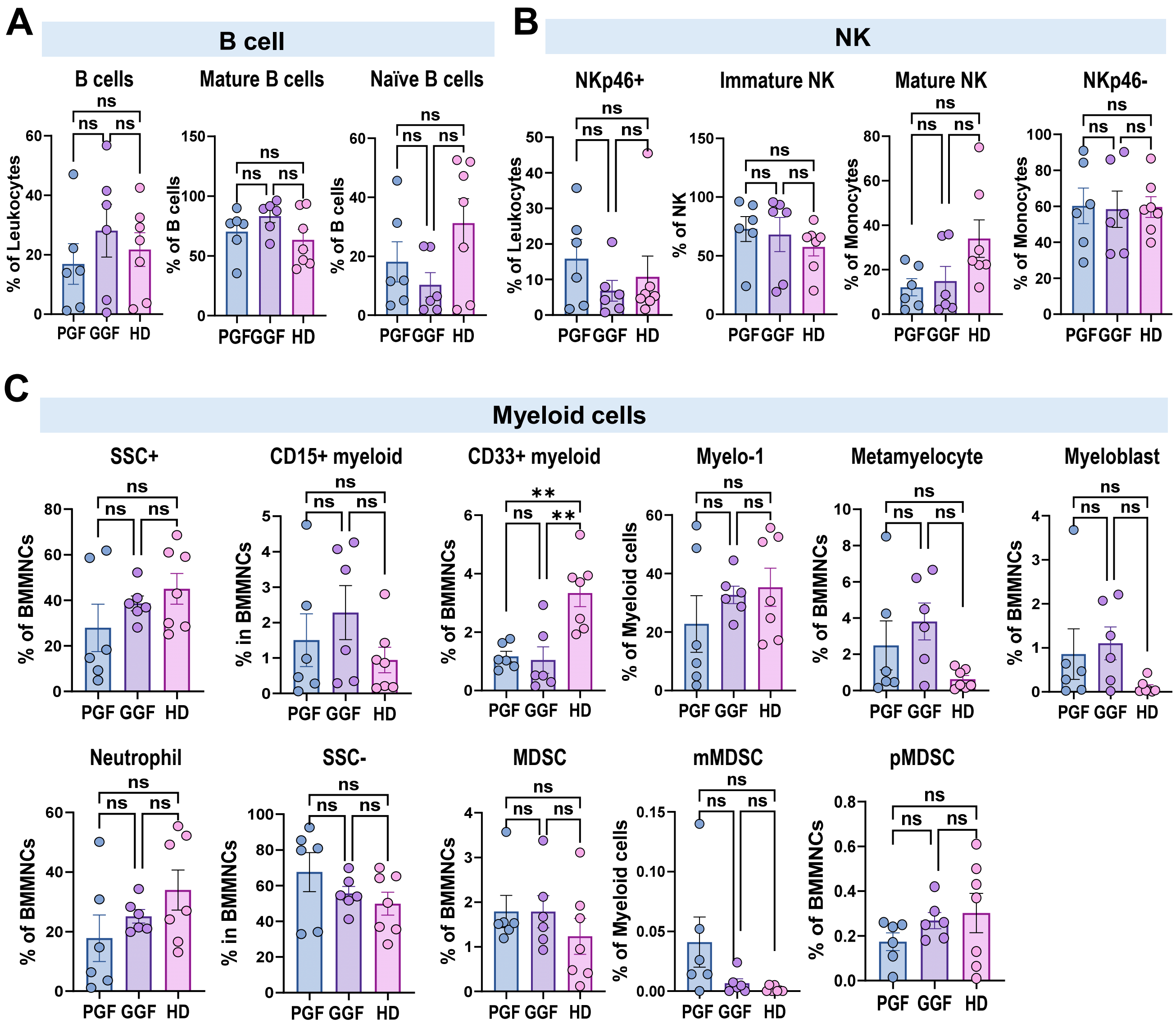

Figure S1. Immunoprofiling of BM samples (related to Figure 2)

Flow cytometry was performed to profile major cell populations in BM.

Percentages of B cells (A), NK cells (B), myeloid cells (C), and their subpopulations were compared among PGF (n = 6), GGF (n = 6) patients, and HDs (n = 7).

Statistical analysis was performed using the ordinary one-way ANOVA test and these data are shown with mean values ± SEM. *, $p < 0.05$; **, $p < 0.01$; ***, $p < 0.001$; ****, $p < 0.0001$; ns, no statistical significance.

BM, bone marrow; NK cell, natural killer cell; PGF, poor graft function; GGF, good graft function; HDs, healthy donors; NK cell, natural killer cell; SSC+, side scatter positive; SSC-, side scatter negative; MDSC, myeloid-derived suppressor cell; mMDSC, monocytic MDSC; pMDSC, polymorphonuclear MDSC; SEM, standard error of the mean.

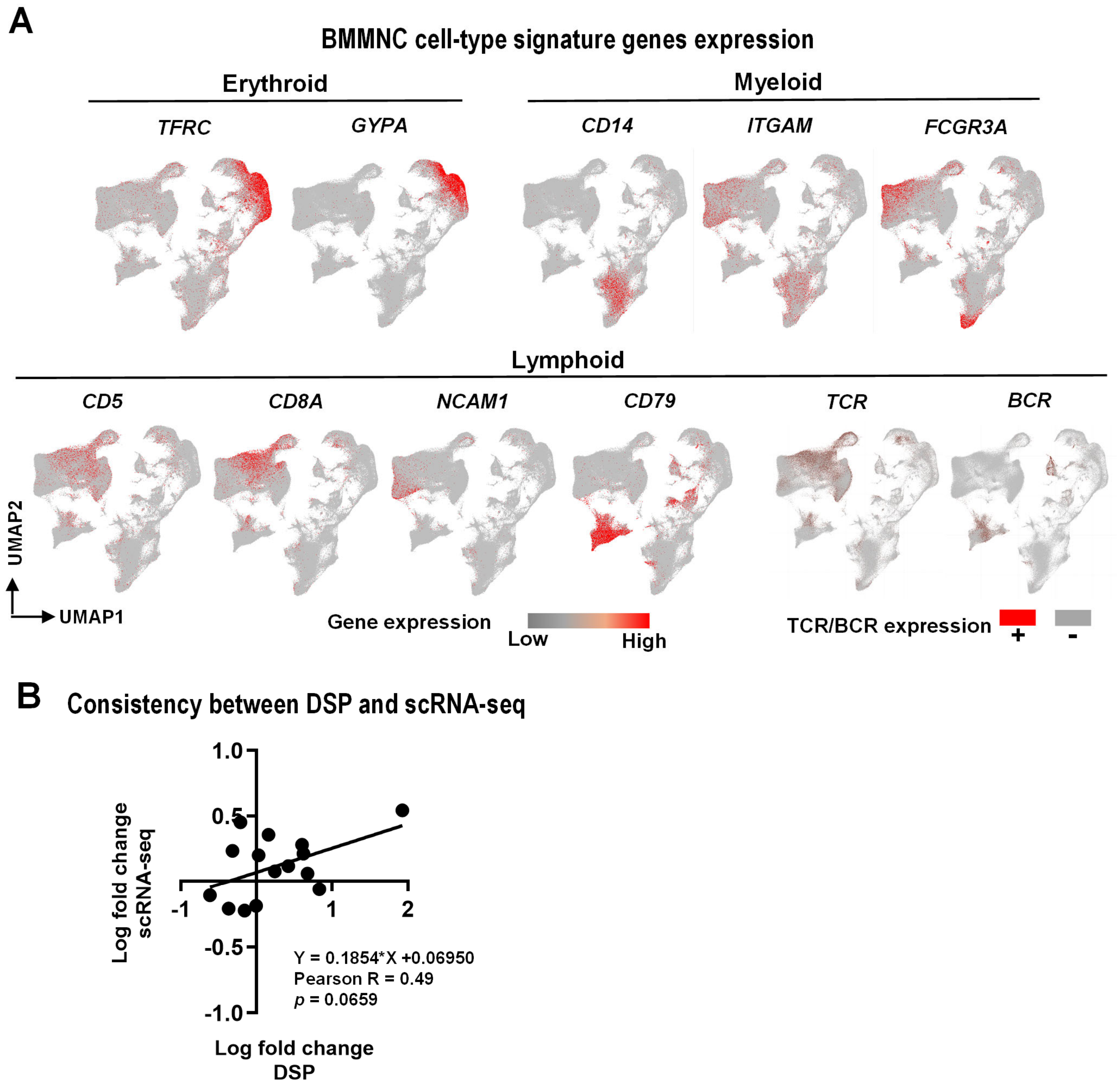

Figure S2. scRNA-seq and DSP of BM samples (related to Figure 3)
(A) Expression of lineage signature genes, cell-type specific genes, and TCR/BCR are highlighted in UMAP lots of batch-corrected single-cell gene expression in BMMNCs of all samples: the same UMAP plot in Figure 3A.
(B) Correlation of log fold changes in scRNA-seq and DSP were analyzed. A *p* value and a slope with the Pearson correlation test are shown.
scRNA-seq, single-cell RNA sequencing; DSP, digital spatial profiling; TCR/BCR, T cell receptor/B cell receptor; UMAP, Uniform Manifold Approximation and Projection; BMMNCs, bone marrow mononuclear cell.

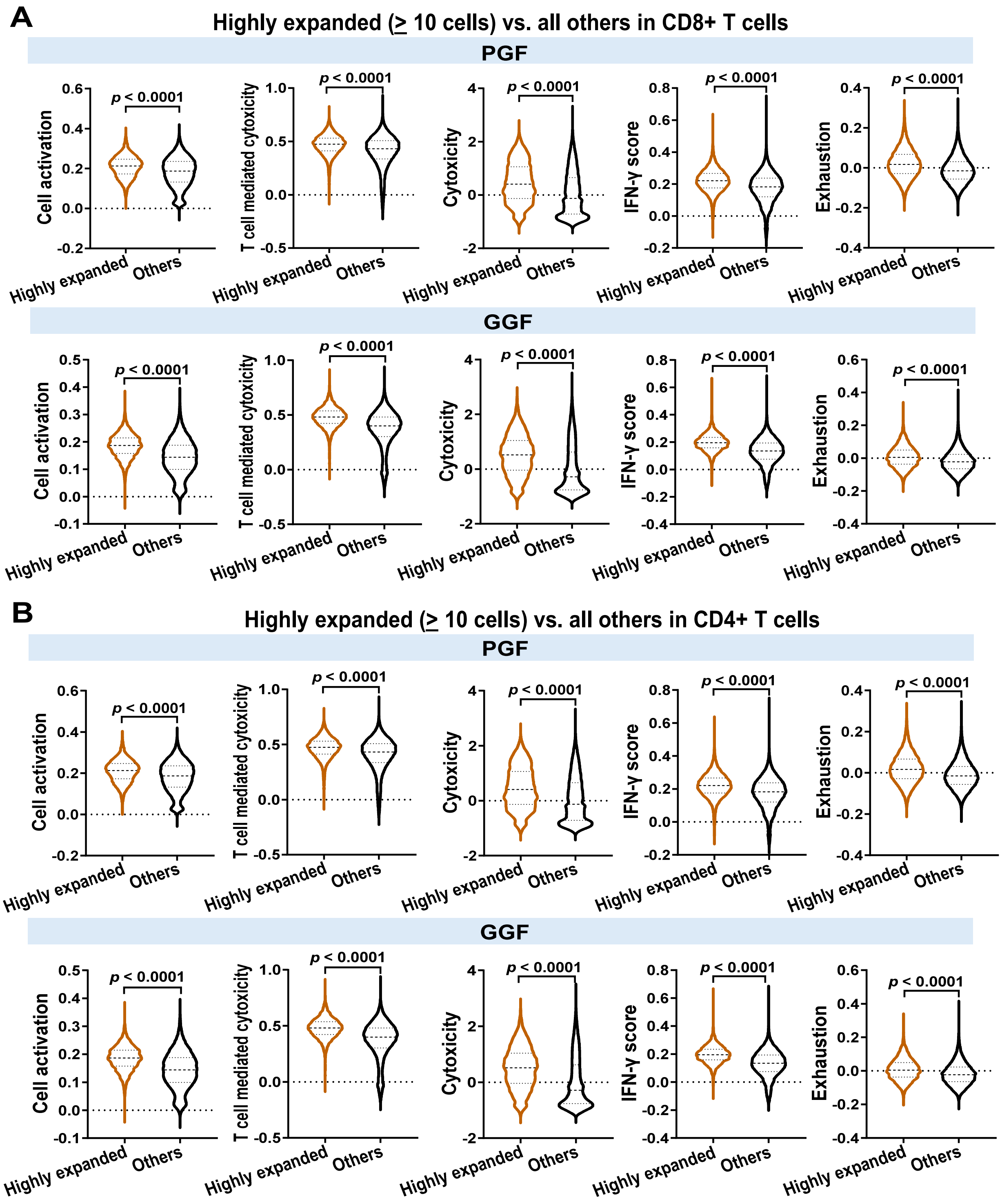

Figure S3. Clonally expanded T cells are activated in PGF and GGF after transplantation (related to Figure 5)
(A) Relative inflammatory pathway (cell activation, T cell mediated cytotoxicity, cytotoxicity, IFN-γ signaling, and exhaustion) scores of highly expanded cells were compared with other cells in CD8^{+} T cells (A) or CD4^{+} T cells (B) evaluated by scRNA-seq in PGF (n = 6) and GGF (n = 5).
p values of the ordinary one-way ANOVA test are shown. *, $p < 0.05$; **, $p < 0.01$; ***, $p < 0.001$; ****, $p < 0.0001$; ns, no statistical significance.

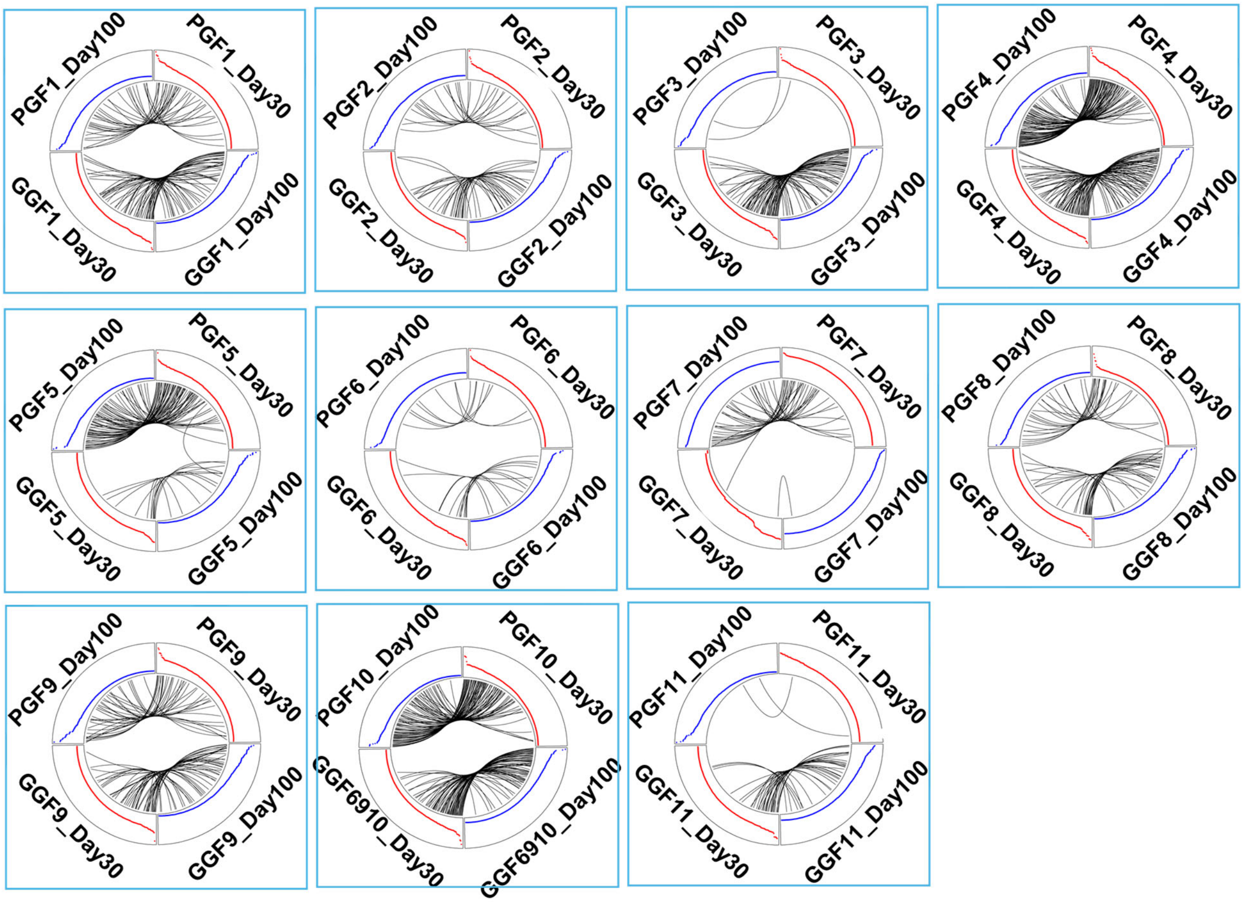

Figure S4. TCR usage are shared at different timepoints in the same individual, but minimal across individuals (related to Figure 6)

Circos plots are shown segments in circles represent individual cells yielding rearranged TCR sequences. Black lines connect clones sharing identical CDR3 sequences among individuals. Sharing of identical CDR3 sequences between PGF patients (day 30 and day 100) and matched GGF patients (day 30 and day 100). Red and blue curves are proportional to clone sizes.

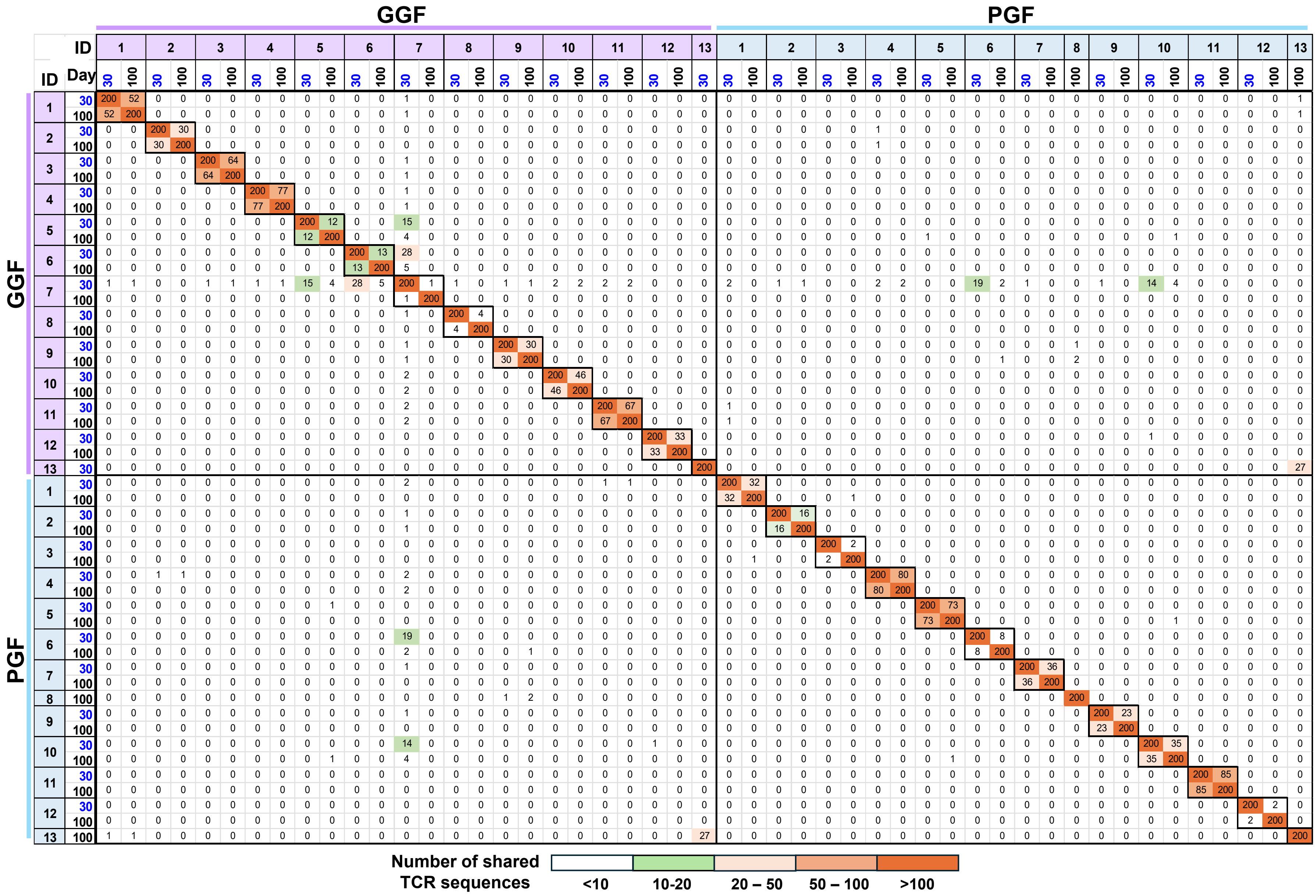

Figure S5. A heatmap plot showing the number of common TCR clones in PGF GGF samples (related to Figure 6)

The number of common TCR clones in PGF (n = 11) and GGF (n = 12) samples were examined among top 200 TCR clones. Both x-and y-axes represent samples of patients. Samples at different timepoints (day 30 and day 100) of the same patients were adjacent. Numbers indicate counts of identical TCR clones shared among samples. A color scheme ranging from brown to white represents the number of shared CDR sequences from high to low. In general, there was lack of common TCR usage in PGF and GGF patients after transplantation.

Supplementary Information

T cell and monocyte activation in concert with hematopoietic stem cell interactions shapes the post-allogeneic transplant immune landscape in poor graft function

Short title: multiomic evaluation of hematopoiesis and immune landscape post-allogeneic stem cell transplantation

Keywords: hematopoietic cell transplantation; single-cell RNA sequence; T cell receptor; immune cell interactions; immune reconstitution

Ashvind Prabahran,[1,2,3,4,]* Zhijie Wu,[4,]* Shouguo Gao,[4] Huw Morgan,[2] Nicholas Holzwart,[2] Mandy Ludford-Menting,[2] Mayani Rawicki,[2] Jessica Klass[1,2] Ray-Mun Koo,[1,2,3] Clarissa Wilson,[5] Piers Blombery,[5] Chin Wee Tan,[6] Saanvi Indukuri,[4] Lynette Chee,[1,2,3] David Ritchie,[1,2,3] Neal S. Young,[4] Xingmin Feng,[4,†] and Rachel Koldej,[2,3,†]

*A.P. and Z.W. contributed equally to this work.

[†]co-last authors

Supplementary Methods

Key resource table

REAGENT or RESOURCE	SOURCE	IDENTIFIER
Antibodies		
Anti-human lineage cocktail (Clones UCHT1, HCD14, 3G8, HIB19, 2H7, and HCD56) Pacific blue	BioLegend	Cat# 348805; RRID: AB_2889063
Mouse anti-human CD34 (Clone 581) PE	BD Biosciences	Cat# 555822; RRID: AB_396151
Mouse anti-human CD38 (Clone HIT2) APC	BD Biosciences	Cat# 555462, RRID: AB_398599
Mouse anti-human CD90 (Clone 5E10) FITC	BioLegend	Cat# 328108, RRID: AB_893429
Mouse anti-human CD10 (Clone HI10A) BV605	BD Biosciences	Cat# 562978, RRID: AB_2737929
Mouse anti-human CD135 (Clone BV10A4H2) PE/Cyanine7	BioLegend	Cat# 313314, RRID: AB_2565478
Mouse anti-human CD45RA (Clone HI100) BV510	BioLegend	Cat# 304142, RRID: AB_2561947
Mouse anti-human CD3 (Clone UCHT1) Pacific Blue	BioLegend	Cat# 980004, RRID: AB_324164
Mouse anti-human CD14 (Clone HCD14) Pacific Blue	BioLegend	Cat# 325616, RRID: AB_830689
Mouse anti-human CD16 (Clone 3G8) Pacific Blue	BioLegend	Cat# 302032, RRID: AB_ 2104003
Mouse anti-human CD19 (Clone HIB19) Pacific Blue	Biolegend	Cat# 302232, RRID: AB_ 2073118
Mouse anti-human CD56 (Clone HCD56) Pacific Blue	BioLegend	Cat# 318326, RRID: AB_ 10612566
Mouse anti-human CD20 (Clone 2H7) Pacific Blue	BioLegend	Cat# 302328, RRID: AB_ 493651
Mouse anti-human PD-1 (Clone A17188B) FITC	BioLegend	Cat# 621612, RRID: AB_ 2832832
Mouse anti-human CD57 (Clone HCD57) APC	BioLegend	The product has been discontinued.
Mouse anti-human CD3 (Clone UCHT1) BV711	BD	Cat# 563725, RRID: AB_ 2744392
Mouse anti-human CD38 (Clone HB-7) PE	BioLegend	Cat# 356604, RRID: AB_ 2561899
Mouse anti-human CD25 (Clone M-A251) PE-Cy7	BD	Cat# 560920, RRID: AB_ 396847
Mouse anti-human HLA-DR (Clone L243) APC-Cy7	BioLegend	Cat# 307618, RRID: AB_ 396847
Mouse anti-human CD8 (Clone RPA-T8) Pacific Blue	BioLegend	Cat# 301033, RRID: AB_ 393111
Mouse anti-Human CD4 (Clone SK3) BUV496	BD	Cat# 612936, RRID: AB_ 2028488
Mouse anti-human CD127 (Clone HIL-7R-M21)	BD	Cat# 557938, RRID: AB_ 2296056

Mouse anti-human CD95 (Clone DX2) BV786	BioLegend	Cat# 305646, RRID: AB_2629742
Mouse anti-human CD45RA (Clone HI100) BV510	BioLegend	Cat# 304142, RRID: AB_2561947
Mouse anti-human CCR7 (Clone G943H7) Alexa700	BioLegend	Cat# 353244, RRID: AB_2617001
Mouse anti-human PD-L1 (Clone MIH1) BUV661	BD	Cat# 741666, RRID: AB_2871057
Mouse anti-human PD-2 (Clone MIH18) BUV661	BD	Cat# 755317, RRID: AB_2162177
Mouse anti-human 4-1BB (Clone 4B4-1) BUV737	BD Optibuild	Cat# 749141, RRID: AB_1272083
Mouse anti-human PDL1 (Clone MIH1) BV421	BD horizon	Cat# 563738, RRID: AB_2738396
Anti-human ICOS (Clone ISA-3) SB436	Invitrogen	Cat# 62-9948-42, RRID: AB_2637393
Anti-human TCRgd (Clone 11F2) BV650	BD Optibuild	Cat# 745359, RRID: AB_2742924
Mouse anti-human CD123 (Clone 9F5) BV750	BD Optibuild	Cat# 747136, RRID: AB_2871883
Mouse anti-human PD-1 (CloneEH12.1) BB515	BD Horizon	Cat# 565936, RRID: AB_2738827
Mouse anti-human CD15 (Clone HI98) PerCP eFluor 710	Invitrogen	Cat# 46-0159-42, RRID: AB_1834387
Mouse anti-human TIM3 (Clone F38-2E2) PE eFluor 610	Invitrogen	Cat# 61-3109-42, RRID: AB_2802391
Mouse anti-human LAG-3 (Clone T47-530) AlexaFluor 647	BD Pharmingen	Cat# 565716, RRID: AB_2744328
Anti-human CD56 (Clone B159) AlexaFluor	BD Pharmingen	Cat# 557699, RRID: AB_396808
Mouse anti-human CD127 (Clone eBioRDR5) APC eFluor	Invitrogen	Cat# 47-1278-42, RRID: AB_1548674
Anti-Human HLA- ABC (Clone G46.6) BUV395	BD Horizon	Cat# 564040, RRID: AB_2738558
Mouse anti-human CD14 (Clone M5E2) BUV496	BD Optibuild	Cat# 750381, RRID: AB_2874552
Mouse anti-human CD5 (Clone UCHT2) BUV563	BD Optibuild	Cat# 741354, RRID: AB_2870855
Mouse anti-human CD4 (Clone SK3) BUV805	BD Horizon	Cat# 612888, RRID: AB_2870177
Mouse anti-human 11b (Clone ICRF44) Pac Blue	Bio Legend	Cat# 301315, RRID: AB_493016
Mouse anti-human CD8 (Clone RPA-T8) BV480	BD Horizon	Cat# 566121, RRID: AB_2739523
Mouse anti-human CD3 (Clone OKT-3) BV510	Bio Legend	Cat# 317332, RRID: AB_2561376
Mouse anti-human CD16 (Clone 3G8) BV570	Bio Legend	Cat# 302036, RRID: AB_10915988
Mouse anti-human CD62L (Clone DREG-56) BV605	Bio Legend	Cat# 304833, RRID: AB_2562129
Mouse anti-human CD163 (Clone GHI/6I) BV711	Bio Legend	Cat# 333630, RRID: AB_2650971

Mouse anti-human HLA-DR (Clone G46-6) BV786	Bio Legend	Cat# 564041, RRID: AB_2738559
Mouse anti-human CD45RA (Clone HI100) PerCP Cy5.5	Invitrogen	Cat# 45-0458-42, RRID: AB_10718536
Mouse anti-human CCR7 (Clone 150503) PE	BD Pharmingen	Cat# 560765, RRID: AB_2033949
Mouse anti-human CD11c (Clone B-Ly6) PE Cy5	BD Pharmingen	Cat# 551077, RRID: AB_394034
Mouse anti-human CD33 (Clone P67.6) PE Cy7	BD	Cat# 333946, RRID: AB_399961
Mouse anti-human CD19 (Clone SJ25C1) APC	BD	Cat# 561743, RRID: AB_396873
Mouse anti-human CD66b (Clone 6/40c) PE-Fire 640	Bio Legend	Cat# 392918, RRID: AB_2876709
Anti-human STING (Clone T3-680) BV421	BD Horizon	Cat# 564966, RRID: AB_2739027
Mouse anti-human VISTA (Clone mih65.RMab) BUV737	BD Optibuild	Cat# 749648, RRID: AB_2873924
Reagents for Digital Spatial Profiling		
Mouse anti-human CD3E (Clone UMAB54)	Origene Technologies	SKU UM500048
AF647 antibody labeling Kit	ThermoFisher	S30044
AF594 antibody labeling Kit	ThermoFisher	A30008
Rabbit anti-human CD45 (Clone D9M81)	Cell Signaling Technology	13917S
Syto83 Nucleic Acid Stain	ThermoFisher Scientific	S11364
Biological samples		
10% fetal bovine serum	Sigma-Aldrich	Cat# 12306C
Healthy bone marrow sample	National Institutes of Health	Healthy bone marrow sample
Patients' bone marrow sample	The Royal Melbourne Hospital	Patients' bone marrow sample
Patients' peripheral blood sample	The Royal Melbourne Hospital	Patients' peripheral sample
Healthy peripheral blood sample	The Royal Melbourne Hospital	Healthy peripheral blood sample
Chemicals, peptides, and recombinant proteins		
LSM Lymphocyte Separation Medium	MP Biomedicals	Cat# 50494X
Phosphate buffered saline	Lonza	Cat# 17-516Q
ACK lysing buffer	Quality Biological	Cat# 118-156-101
IMDM	Thermo Fisher Scientific	Cat# 12440053
DMSO	Sigma-Aldrich	Cat# 67-68-5
RPMI 1640	Thermo Fisher Scientific	Cat# 11875093
Polybrene	Sigma-Aldrich	Cat# TR-1003
Critical commercial assays		
10x Genomics Single Cell Immune Profiling Solution v2	10x Genomics	Cat# 1000263

**Deposited data**		
Raw and analyzed data	This paper	GEO: GSE293012
Published T-cell receptor sequences in T-LGLL patients and healthy donors	Compare TCR usage with published data Gao, et al. 2022[1]	GEO: GSE168859
Single-cell landscape of hematopoiesis and immunological responses in patients with severe aplastic anemia treated with immunosuppression and eltrombopag	BMMNCs of healthy donors as controls Wu, et al. 2025 (in press)[2]	GEO: GSE247531
**Software and algorithms**		
cellranger count version 7.0.0	10x Genomics	https://www.10xgenomics.com/support/software/cell-ranger/downloads/
cellranger vdj version 7.0.0	10x Genomics	https://www.10xgenomics.com/support/software/cell-ranger/downloads/
Seurat version 4.3.0	Stuart et al., 2019[3]	https://cran.r-project.org/web/packages/Seurat/index.html
R version 4.2.2	R Core Team, 2021[4]	https://www.r-project.org/
CellPhoneDB version 3.1.0	Garcia-Alonso L, et al. 2022[5,6]	https://www.cellphonedb.org/
fGSEA version 1.16.0	Korotkevich et al., 2019[7]	http://bioconductor.org/packages/release/bioc/html/fgsea.html
AUCell version 1.4.1	Aibar et al., 2017[8]	https://bioconductor.org/packages/release/bioc/html/AUCell.html
tCR version 2.3.2	Github repository	https://imminfo.github.io/tcr/
GraphPad Prism 10.2.1	GraphPad software	https://www.graphpad.com/scientificsoftware/prism/
FlowJo v7.6.4	Tree Star	https://www.flowjo.com

Bone marrow and peripheral blood samples processing

PBMCs and BMMNCs were isolated and cryopreserved until used for flow cytometric and/or scRNA-seq. PB and BM specimens were obtained from patients and healthy donors and kept in heparin tubes before processing. PBMCs and BMMNCs from each person were isolated by density centrifugation using LSM Lymphocyte Separation Medium (Cat# 50494X, MP Biomedicals). Briefly, PB and BM were diluted twofold using phosphate buffered saline (PBS) (Cat# 17-516Q,

Lonza), layered on top of 1 volume LSM Lymphocyte Separation Medium in a 50-ml Falcon tube, and spun down at 1140g for 25 min at room temperature with brake off. A PBMC or BMMNC layer was isolated and washed with PBS after red blood cell lysing with ACK lysing buffer (Cat# 118-156-101, Quality Biological). PBMCs and BMMNCs were cryopreserved in RPMI 1640 (Cat# 11875093) + fetal bovine serum (Cat# 12306C, Sigma-Aldrich) + 30% DMSO (Cat# 67-68-5, Sigma-Aldrich). Cryopreserved PBMCs and BMMNCs were thawed before multi-color flow cytometry and scRNA-seq.

Digital Spatial Profiling

Bone marrow trephine samples were sectioned at 4µm thickness and mounted on SuperFrost slides. Two trephine sections were mounted per slide. Region of Interest (ROI) selection was undertaken in the ACRF translational research laboratory. The GeoMX DSP kit was used to perform DSP as per manufacturer recommendations. Briefly, a multiplexed cocktail of primary antibodies conjugated to a UV-photocleavable indexing oligo, CD3 antibody, CD45 antibody was applied to a slide mounted BM-trephine section. A pre-designed GeoMX™ DSP panel was applied to each region to determine the expression of 57 proteins (4-1BB, ARG1, B7-H3, BCL2, Beta-2-Microglobulin, CD11c, CD127, CD14, CD163, CD20, CD25, CD27, CD3, CD34, CD4, CD40, CD44, CD45, CD45RO, CD56, CD66b, CD68, CD8, CD80, CTLA4, EpCAM, ERa, FAPa, Fibronectin, FOXP3, GAPDH, GITR, GZMB, Her2, Histone H3, HLA-DR, ICOS, IDO1, Ki-67, LAG-3, MART1, Ms IgG1, Ms IgG2a, NY-ESO-1, OX40L, PanCK, PD-1, PD-L1, PD-L2, PTEN, Rb IgG1, S100B, S6, SMA, STING, TIM-3, VISTA). Regions of interest were selected based on presence of dual CD3/CD45 staining. Six ROIs of 300µm circles were selected. ROIs were processed sequentially by focusing UV light through each ROI and the released oligonucleotides

were aspirated. Indexing oligos were hybridized to Nanostring optical barcodes for ex-situ digital counting and subsequently analyzed with nCounter Analysis System.

Bioinformatics Pipeline for DSP Analysis

The bioinformatics pipeline including data exploration and quality checks, differential expression analyses have been detailed previously. Briefly, following data exploration and quality checks on raw data, raw counts were normalized to the ERCC positive controls and then by trimmed mean of M-Values. Differential expression analysis was undertaken using the R/Bioconductor package Limma (v3.44.3). The main co variates explored in this analysis were disease state from which two comparisons were modelled: PGF versus GGF,.

Flow cytometry profiling of PBMCs

Flow cytometry on peripheral blood samples was performed at the ACRF laboratory. Cryopreserved peripheral blood samples obtained at specified study timepoints were thawed and used to characterize T-cell, NK cell, myeloid and dendritic cell subsets as well as activating and inhibitory receptor expression. After thawing cells were resuspended in FACS buffer (PBS+2% FBS). Cells were stained with Live/Dead aqua (Thermo Fisher) for 30 minutes at 4°C, washed in FACS buffer (2% FBS in PBS) followed by Fc Block (BD Biosciences) and CellBlox Blocking Buffer (Thermo Fisher) for 10 minutes at RT. Cells were stained with NovaRed685-antiCD25 for 30 minutes at 4°C followed by remaining surface antibodies for a further 30minutes at 4°C. After washing twice with FACS buffer, cells were permeabilized using Cytofix/Cytosperm kit (BD sciences) according to instructions. Cells were stained with BV421-antiSTING in Perm/wash for 30 minutes at 4°C prior to 2 washes with Per/Wash buffer. Samples were resuspended in FACS

buffer and acquisition performed with Aurora spectral flow cytometer (Cytek). Analysis was performed using FlowJo (BD Bioscience) software.

Flow cytometry profiling of BMMNCs

Flow cytometry on 12 bone marrow samples was performed at the Hematopoiesis and Bone Marrow Failure Laboratory at the National Heart, Lung, and Blood Institute (NHLBI) Bethesda MD as part of a collaboration. After thawing bone marrow samples were thawed and used to characterize T, NK, B, myeloid and HSPC subsets. Antibodies used listed in Table above. Staining protocols were as for PBMC samples. Cells acquisition was performed on a BD Fortessa Flow Cytometer and analyzed using FlowJo (BD Bioscience) Software.

For HSPC subpopulation profiling, BMMNCs were stained with antibody mixtures on ice for 30 min in RPMI 1640 (Cat# 11875093, Thermo Fisher Scientific). Samples were subsequently acquired using the BD LSR Fortessa cytometer (BD Biosciences), and post-acquisition analysis was performed using Flowjo software (v.7.6.4; Flowjo LLC, BD Biosciences). Antibodies used for flow cytometry analyses were: anti-human lineage cocktail (CD3, CD14, CD16, CD19, CD20, and CD56; clones UCHT1, HCD14, 3G8, HIB19, 2H7, and HCD56, Cat# 348805, BioLegend) in Pacific Blue; anti-human CD34 in PE (clone 581, Cat# 550761, BD Biosciences), anti-human CD38 in APC (clone HIT2, Cat# 555462, BD Biosciences), anti-CD90 in FITC (clone 5E10, Cat# 328108, BioLegend), anti-human CD10 in BV605 (clone HI10A, Cat# 562978, BD Biosciences), anti-human CD135 in PE/Cy7 (clone BV10A4H2, Cat# 313314, BioLegend), and anti-human CD45RA in BV510 (clone HI100, Cat# 304142, BioLegend). Gating strategies for different cell types and subpopulations were shown below.

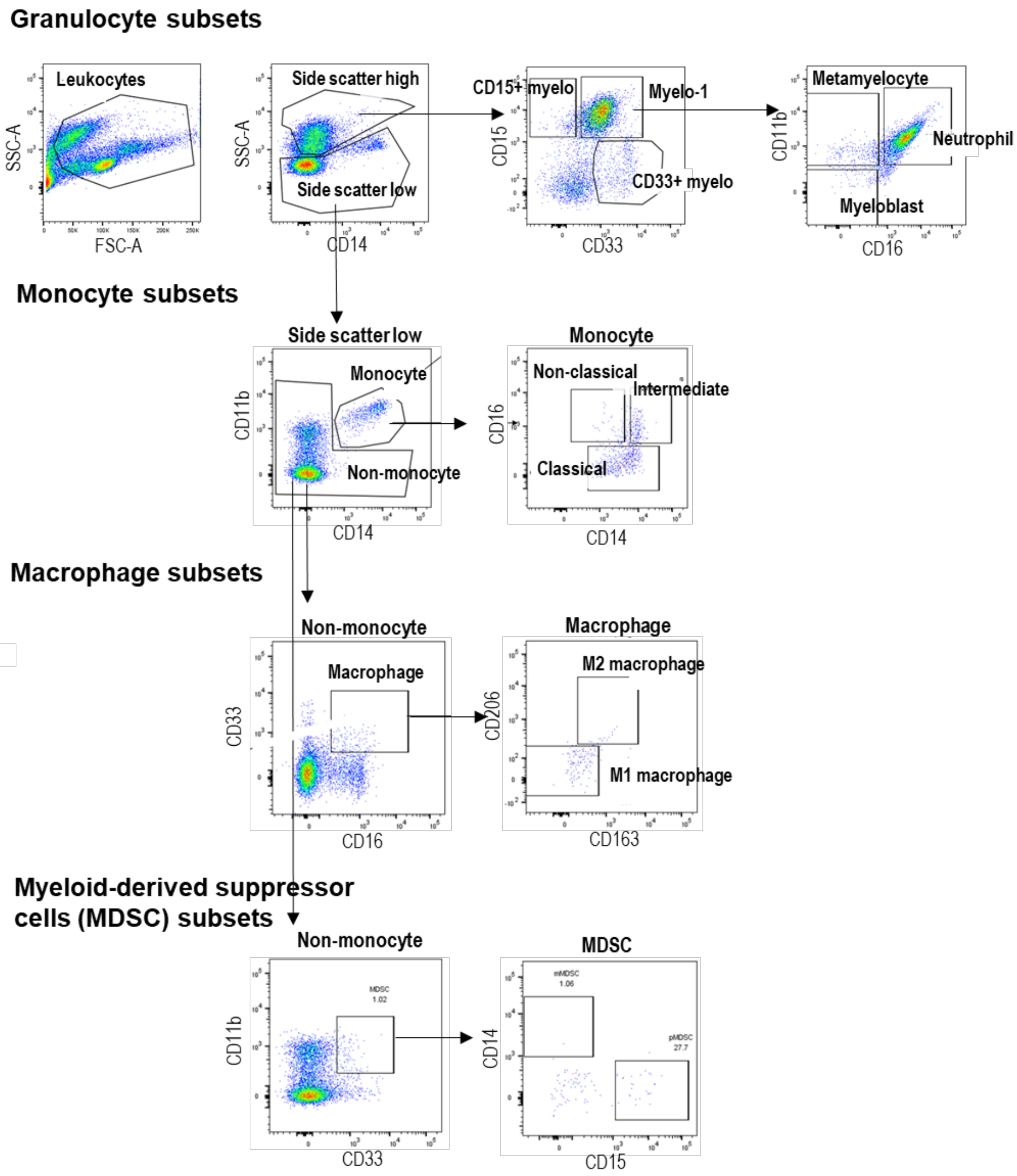
Granulocyte subsets
Leukocytes
SSC-A
FSC-A
Side scatter high
Side scatter low
SSC-A
CD14
CD15+ myelo
Myelo-1
CD33+ myelo
CD15
CD33
Metamyelocyte
Neutrophil
Myeloblast
CD11b
CD16
Monocyte subsets
Side scatter low
Monocyte
Non-monocyte
CD11b
CD14
Monocyte
Non-classical
Intermediate
Classical
CD16
CD14
Macrophage subsets
Non-monocyte
Macrophage
CD33
CD16
Macrophage
M2 macrophage
M1 macrophage
CD206
CD163
Myeloid-derived suppressor cells (MDSC) subsets
Non-monocyte
MDSC
1.02
CD11b
CD33
MDSC
mMDSC
1.06
pMDSC
27.7
CD14
CD15

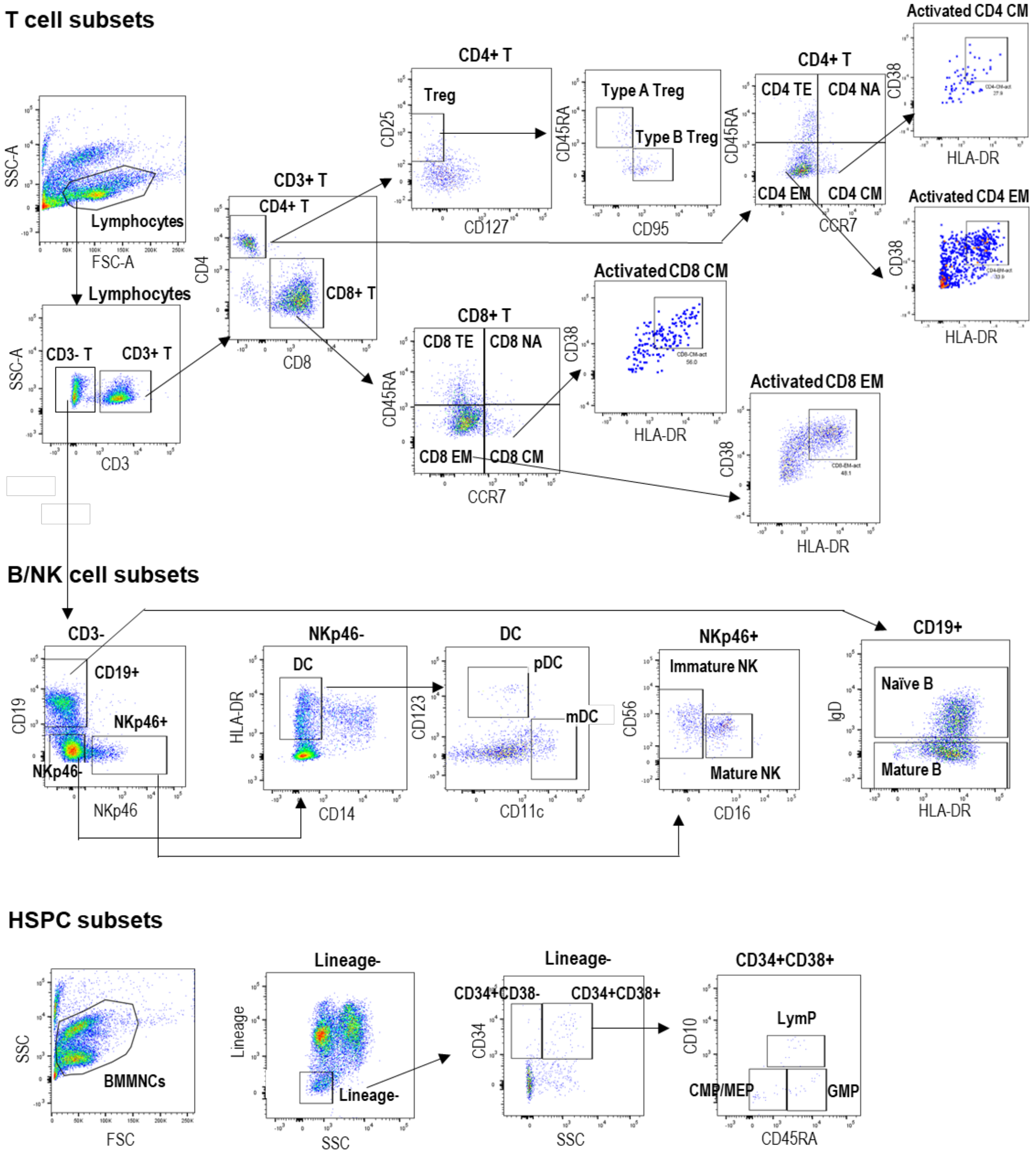

Statistical Analysis of flow cytometry data

Flow cytometry data was evaluated in Graph Pad Prism utilizing a Kruskal-Wallis one-way ANOVA.

Peripheral Blood TCR sequencing

Archival DNA from fractionated CD3+ peripheral blood cells remaining post clinical testing from all patients at D100 post alloSCT was used to perform the analysis. TCRB sequencing was performed using lympho Track TRB as per manufacturer's instructions. MIiXCR was used for sequence assembly from FASTQs, annotation and error correction.

Cell preparation, whole transcriptome amplification (WTA), cDNA library preparation, and sequencing

scRNA-seq coupled with single-cell T cell receptor/B cell receptor sequencing (scTCR/BCR-seq) analysis for patients and healthy donors was performed with the 10x Genomics System using the 10x Genomics Single Cell Immune Profiling Solution v 2 (Chromium Single Cell 5' Reagent Kit v1.1, Cat# 1000263, 10x Genomics), following the manufacturer's protocol (www.10xgenomics.com).[8] Briefly, BMMNCs were washed with 1X PBS with 0.04% (wt/vol) bovine serum albumin. Cell concentration and viability were determined using the Countess II Automatic Cell Counter and the trypan blue staining method. Cell loading and capturing were done on the Chromium Controller (10x Genomics). Following reverse transcription and cell barcoding in droplets, emulsions were broken, and cDNA was purified using Dynabeads MyOne SILANE, followed by PCR amplification. Amplified cDNA was then used for both 3' and 5' gene expression library construction and TCR/BCR enrichment. For gene expression library construction, the amplified cDNA was fragmented, end-repaired, and double-sided size-selected with SPRIselect beads. For TCR/BCR library construction, TCR/BCR transcripts were enriched from amplified cDNA by PCR. Subsequently, the enriched PCR product was fragmented, end-repaired, and size-selected with SPRIselect beads. The scRNA libraries were pooled together and sequenced on the Illumina NovaSeq system using read lengths of 26-bp read 1, 8 bp i7 index, 98-bp read 2. The

single-cell TCR/BCR libraries were sequenced on the Illumina NovaSeq system using read lengths of 150-bp read 1, 8 bp i7 index, 150-bp read 2.

scRNA-seq data analysis

Preprocessing of scRNA-seq and scTCR/BCR-seq data

Alignment, barcode assignment, and Unique Molecular Identifier (UMI) counting were performed using the cellranger pipeline[9] (http: //software.10xgenomics.com/single-cell/overview/welcome).

After single-cell libraries were sequenced using the Illumina system, cellranger pipeline (https://support.10xgenomics.com/single-cell-gene-expression/software/pipelines/latest/what-is-cell-ranger) was used to process scRNA-seq raw data to align reads to the genome, and to generate gene–cell expression matrices. Specifically, sequencing reads were aligned to the GRCh38 reference genome by STAR with annotation of ENSEMBL. Uniquely aligned reads were used to quantify gene expression levels for ENSEMBL genes with UMIs.

Downstream analyses were performed using the R software[4] package of Seurat (Stuart et al., 2019; http://satijalab.org/seurat/, v4.2.2).[3] UMIs in each cell were first scaled by a library size to 10000, and then log-transformed. To improve downstream dimensionality reduction and clustering, FindIntegrationAnchors and IntegrateData functions were used to integrate scRNA-seq data of all samples. Top 2000 highly variable genes were used for Principal Component Analysis (PCA) of high-dimensional data. Top 50 principal components were selected for unsupervised clustering of cells with a graph-based clustering approach.

TCR reads were aligned to the GRCh38 reference genome and consensus TCR annotation was performed using the cellranger vdj program (10x Genomics, version 3.0.1). TCR annotation was performed using the 10x cellranger vdj pipeline as described at https://support.10xgenomics.com/single-cell-vdj/software/pipelines/latest/using/vdj. Barcodes

with a higher number of UMI counts more that of simulated background were considered as cell barcodes. V(D)J read filtering and assembly were implemented as a previous study.[10] cellranger firstly trimmed known adaptor and primer sequences from the 5′ and 3' ends of reads, and then filtered away reads lacking at least one 15-bp exact match against at least one reference segment (TCR, TRA, and TRB gene annotations in Ensembl version 87). Next, cellranger performed de novo assembly for each barcode by building a De Bruijn graph of reads independently. Finally, each assembled contig was aligned against all of the germline segment reference sequences of the V, D, J, C, and 5' UTR regions. cellranger searched a CDR3 motif (Cys-to-FGXG/WGXG) in a frame defined by a start codon in the L+V region or all 6 frames when the L+V region was absent. Most cell barcodes contained two matching productive contigs, comprising either a TCRA or a TCRB though it was of biological possibility that fewer productive contigs (low sensitivity) or > 2 productive contigs (some cells do contain more than one TCRB or TCRA chain) were associated with one cell barcode.[11] Similarly, BCR reads were also processed using the cellranger vdj program, with the IMGT database of GRCh38 genome as reference. Only productive contigs of BCR were kept for analysis.

Downstream analysis

Dimensionality reduction and clustering were performed by PCA and visualized with Uniform Manifold Approximation and Projection (UMAP). Cell type identity was assigned to each cluster based on significance in overlap between signature genes of BMMNCs[12] and cluster-specific genes (Fisher's exact test). Gene Set Enrichment Analysis (GSEA; http://software.broadinstitute.org/gsea) was used to interpret gene set enrichment and pathways of defined differentially expressed genes. A scoring algorithm to calculate interaction scores,[13]

CellPhoneDB[5,6] was used to examine ligand-receptor interactions.

Comparison of lineage gene Area Under the Receiver Operating Characteristic Curve (AUC) scores

We calculated AUC scores of lineage-specific gene expression (myeloid, lymphoid and erythroid) of single cells in individual patients, and average AUC scores[8] of specific lineages of all cells in each patient were compared. Comparison between two groups was performed using Prism (v.7.02; the GraphPad Software), and results were shown as mean ± standard derivation. Statistical analysis was performed using the two-sided unpaired Mann-Whitney test for two groups. $p < 0.05$ was considered statistically significant.

Differential expression of genes and generation of heatmaps

Differentially expressed genes were defined with the FindMarkers function in Seurat, by comparing gene expression in one cell subset with expression in all others. Heatmaps and network visualization were generated with ggplot2 and heatmap2 in the R package.

Gene set enrichment analysis

GSEA is the widely used pathway analysis tool that determines whether pre-defined gene sets show statistically significant, concordant differences between two biological states. GSEA was performed based on fold change of all detected genes.

Cell activation, inflammation, and exhaustion score calculation

Cell activation, inflammation, and exhaustion score were defined based on the published reference gene list,[14,15] and were calculated with the AddModuleScore function built in Seurat.[3]

Ligand receptor analysis

Cell-cell interactions based on the expression of known ligand-receptor pairs in different cell types were calculated using the CellPhone DB version 3.1.0.[5,6] The algorithm was run on log-normalized

expression values for cell populations of BMMNCs with default parameters and no subsampling to identify the enriched ligand-receptor pairs in PGF and GGF patients and healthy controls.

Diversity index calculation

There are many ways of defining the diversity of a population, clonal types in this study, with each method providing a different representation of the number of clones (identical TCR/BCR chains) present (richness) and of their relative frequency (evenness). The Shannon entropy weighs both of these aspects of diversity equally, and it is an intuitive measure whereby the maximum value is determined by a total size of the repertoire. Entropy values decreases with increasing inequality of frequency as a result of clonal expansion. The Shannon entropy in a population of N clones with nucleotide frequency pi is defined by the following equation:

$$H(P) = -\sum_{i=1}^{n} p_i log_2 p_i$$

The Gini coefficient is most often used in economics to measure a country's wealth distribution and has been widely used in diversity assessment of TCRs/BCRs.[16] The Gini coefficient is usually defined mathematically based on the Lorenz curve or Relative mean absolute difference.[17] The Gini coefficient and Shannon entropy for diversity and clonality analysis were calculated with the R package of tCR (https://imminfo.github.io/tcr/).

Statistical analysis

Pearson correlations between interaction scores and inflammatory and cytokine scores were calculated with the R package. Comparison between groups was performed using the GraphPad Prism (v.10.4.1; GraphPad software, La Jolla, CA), and results were shown as mean ± standard error of the mean.

REFERENCES

1. Gao S, Wu Z, Arnold B, et al. Single-cell RNA sequencing coupled to TCR profiling of large granular lymphocyte leukemia T cells. Nature Communications 2022;13(1):1982. DOI: 10.1038/s41467-022-29175-x.
2. Wu Z, Gao S, Feng X, et al. Human autoimmunity at single cell resolution in aplastic anemia before and after effective immunotherapy. Nature Communications 2025;16(1):5048. DOI: 10.1038/s41467-025-60213-6.
3. Stuart T, Butler A, Hoffman P, et al. Comprehensive Integration of Single-Cell Data. Cell 2019;177(7):1888-1902.e21. (In eng). DOI: 10.1016/j.cell.2019.05.031.
4. (2019). RCT. R: A language and environment for statistical computing. R Foundation for Statistical Computing, Vienna, Austria. URL https://www.R-project.org/. 2019.
5. Garcia-Alonso L, Lorenzi V, Mazzeo CI, et al. Single-cell roadmap of human gonadal development. Nature 2022;607(7919):540-547. DOI: 10.1038/s41586-022-04918-4.
6. Efremova M, Vento-Tormo M, Teichmann SA, Vento-Tormo R. CellPhoneDB: inferring cell-cell communication from combined expression of multi-subunit ligand-receptor complexes. Nat Protoc 2020;15(4):1484-1506. (In eng). DOI: 10.1038/s41596-020-0292-x.
7. Korotkevich G, Sukhov V, Budin N, Shpak B, Artyomov MN, Sergushichev A. Fast gene set enrichment analysis. bioRxiv 2021:060012. DOI: 10.1101/060012.
8. Aibar S, González-Blas CB, Moerman T, et al. SCENIC: single-cell regulatory network inference and clustering. Nature Methods 2017;14(11):1083-1086. DOI: 10.1038/nmeth.4463.
9. Zheng GXY, Terry JM, Belgrader P, et al. Massively parallel digital transcriptional profiling of single cells. Nature Communications 2017;8(1):14049. DOI: 10.1038/ncomms14049.
10. Azizi E, Carr AJ, Plitas G, et al. Single-Cell Map of Diverse Immune Phenotypes in the Breast Tumor Microenvironment. Cell 2018;174(5):1293-1308.e36. (In eng). DOI: 10.1016/j.cell.2018.05.060.
11. Redmond D, Poran A, Elemento O. Single-cell TCRseq: paired recovery of entire T-cell alpha and beta chain transcripts in T-cell receptors from single-cell RNAseq. Genome Medicine 2016;8(1):80. DOI: 10.1186/s13073-016-0335-7.
12. Hay SB, Ferchen K, Chetal K, Grimes HL, Salomonis N. The Human Cell Atlas bone marrow single-cell interactive web portal. Exp Hematol 2018;68:51-61. (In eng). DOI: 10.1016/j.exphem.2018.09.004.
13. Kumar MP, Du J, Lagoudas G, et al. Analysis of Single-Cell RNA-Seq Identifies Cell-Cell Communication Associated with Tumor Characteristics. Cell Reports 2018;25(6):1458-1468.e4. DOI: https://doi.org/10.1016/j.celrep.2018.10.047.
14. Liberzon A, Birger C, Thorvaldsdóttir H, Ghandi M, Mesirov JP, Tamayo P. The Molecular Signatures Database (MSigDB) hallmark gene set collection. Cell Syst 2015;1(6):417-425. (In eng). DOI: 10.1016/j.cels.2015.12.004.
15. Saif MW, Hopkins JL, Gore SD. Autoimmune phenomena in patients with myelodysplastic syndromes and chronic myelomonocytic leukemia. Leuk Lymphoma 2002;43(11):2083-92. (In eng). DOI: 10.1080/1042819021000016186.
16. Rosati E, Dowds CM, Liaskou E, Henriksen EKK, Karlsen TH, Franke A. Overview of methodologies for T-cell receptor repertoire analysis. BMC Biotechnology 2017;17(1):61. DOI: 10.1186/s12896-017-0379-9.
17. Oakes T, Heather JM, Best K, et al. Quantitative Characterization of the T Cell Receptor Repertoire of Naïve and Memory Subsets Using an Integrated Experimental and Computational Pipeline Which Is Robust, Economical, and Versatile. Front Immunol 2017;8:1267. (In eng). DOI: 10.3389/fimmu.2017.01267.
